# High-Precision Low-Cost Gimballing Platform for Long-Range Railway Obstacle Detection

**DOI:** 10.3390/s22020474

**Published:** 2022-01-09

**Authors:** Elio Hajj Assaf, Cornelius von Einem, Cesar Cadena, Roland Siegwart, Florian Tschopp

**Affiliations:** 1 Autonomous Systems Lab, ETH Zurich, 8092 Zurich, Switzerland; ehajjassaf@student.ethz.ch (E.H.A.); cesarc@ethz.ch (C.C.); rsiegwart@ethz.ch (R.S.); tschopp@arrival.com (F.T.); 2 Arrival Ltd., London W14 8TS, UK

**Keywords:** pointing mechanism, high precision actuation, long-range obstacle detection

## Abstract

Increasing demand for rail transportation results in denser and more high-speed usage of the existing railway network, making new and more advanced vehicle safety systems necessary. Furthermore, high traveling speeds and the large weights of trains lead to long braking distances—all of which necessitates a Long-Range Obstacle Detection (LROD) system, capable of detecting humans and other objects more than 1000 m in advance. According to current research, only a few sensor modalities are capable of reaching this far and recording sufficiently accurate data to distinguish individual objects. The limitation of these sensors, such as a 1D-Light Detection and Ranging (LiDAR), is however a very narrow Field of View (FoV), making it necessary to use high-precision means of orienting to target them at possible areas of interest. To close this research gap, this paper presents a high-precision pointing mechanism, for the use in a future novel railway obstacle detection system, capable of targeting a 1D-LiDAR at humans or objects at the required distance. This approach addresses the challenges of a low target price, restricted access to high-precision machinery and equipment as well as unique requirements of our target application. By combining established elements from 3D printers and Computer Numerical Control (CNC) machines with a double-hinged lever system, simple and low-cost components are capable of precisely orienting an arbitrary sensor platform. The system’s actual pointing accuracy has been evaluated using a controlled, in-door, long-range experiment. The device was able to demonstrate a precision of 6.179 mdeg, which is at the limit of the measurable precision of the designed experiment.

## 1. Introduction

Existing railway networks are reaching their operational capacities due to rising global demand for rail transportation. Reasons for this include increasing international trade and changing consumer behavior due to raising environmental awareness and changes in personal mobility needs. Consequently, upgrades to the existing modes of operation and railway control systems, in the form of safer, more reliable, and more efficient trains, are required to keep up with the current growth. More precisely, new railway operation modes require not only reliable communication between rail vehicles, and continuous, accurate and robust localization (European Train Control System (ETCS) Level 0–2 [1]) of each train, but also environmental awareness in the form of Long-Range Obstacle Detection (LROD). In particular, the early detection of humans, animals, or objects on, or in the vicinity of the railway tracks can not only prevent life-threatening risks to humans but also extensive disruptions to the operation of the railway network. However, due to the high weight and velocity of rail vehicles, and the lower traction limiting the braking force, long distances are required for safe braking [2]. Given these circumstances, obstacle detection systems need to be capable of reliably detecting and positioning possible dangers at long ranges, greater than 1000 m.

**Figure 1:**
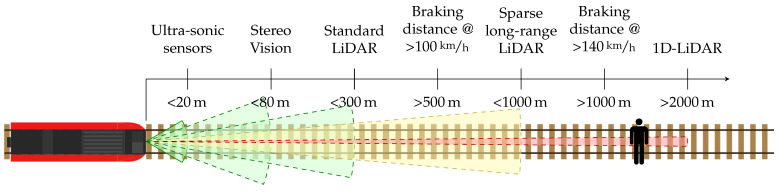
Sensing range of a variety of sensors commonly used for obstacle detection and autonomous vehicles. Furthermore, braking distances at various velocities are shown. The Field of View (FoV) of the individual sensors is highlighted through their color, ranging from super wide FoVs (green) to single point measurements (red).

Several technologies and solutions are already in use for obstacle detection in different applications, for example, in the automotive industry. However, there are several limitations to all of them restricting their direct transferability to the railway domain, as shown in Figure 1. The visualization highlights the long-range requirements for the application in railway systems in comparison to the capabilities of different sensor modalities, such as stereo vision and Light Detection and Ranging (LiDAR). Two additional solutions for detecting humans and other objects, even in challenging situations, are color and thermal cameras [3]. However, the lack of range information makes it difficult to accurately locate the detected obstacle, reducing the system’s reliability. Data driven methods can aid in providing vision based depth information at impressive ranges, but are highly dependent on training data. Stereo camera setups can provide additional depth information to the system [4] by computing the binocular disparity of an object which is observed by both cameras. As the depth resolution is dependent on the stereo baseline (the distance between the two cameras), a baseline larger than the width of the train would be required to achieve accurate enough depth measurements to be reliable for the obstacle detection process.

Additionally, radar sensors are already in use for automotive driver assistance systems and can achieve measurement ranges above 1000 m. As they rely though on the reflection of emitted radio waves from all target objects, they are limited to special radar reflective materials, such as metal, and sufficiently large targets, and are therefore not suited for detecting generic unknown obstacles at such distances.

Currently available dense LiDAR sensors can sample their environment accurately enough for detecting objects, by emitting multiple beams of light and observing their reflections, but are typically limited to a range under 300 m. Sparse long-range LiDAR systems exist [5], though with reduced measurement density and increased measurement times, making them unsuitable for detecting objects while the sensor is moving at high speed.

The limitations of the aforementioned sensor systems make it clear that, in order to achieve an accurate detection and localization of obstacles at long range, it is necessary to integrate various sensor modalities into a complete detection system.

**Figure 2:**
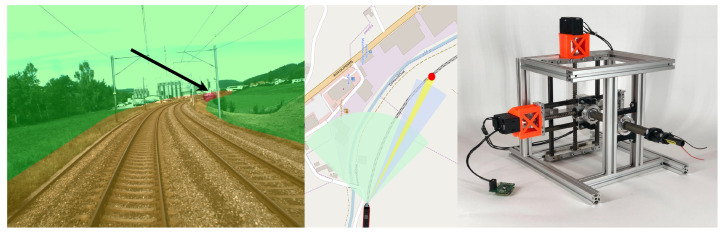
Long-Range Obstacle Detection systems need to focus their sensors on targeted Regions of Interest, shown in yellow and red (arrow), in the vicinity of the railway track. The change of attitude of individual sensors is shown in the centre image, to focus the sensors on possible obstacles. This can be done using a high precision pointing mechanism as shown on the right.

To address these limitations, we propose to develop a combined camera-LiDAR sensor system capable of providing accurate and reliable obstacle detection and positioning at large distances. The proposed system consists of a wide-angle overview camera observing the train’s entire FoV (the tracks and their immediate vicinity lying ahead of the train), as shown in green in Figure 2. Based on the vision system as well as a known track map, a possible Region of Interest (RoI) can be determined (shown in yellow) within which possible obstacles might be located. Additional cameras and LiDAR sensors are then supposed to detect these obstacles at high range within this RoI. 1D-LiDAR sensors exist with multiple kilometers of range. However, these sensors are only capable of measuring a few points per second and can therefore not detect objects on their own. Instead, we propose to couple such a sensor with a high focal length camera to produce an image with combined depth measurement. Due to the very limited FoV of this setup, as well as the long measurement time of the laser, an active vision approach is required. The system needs to be placed on a gimballing platform capable of orienting the combined sensor setup at possible targets located within the specified RoI. This is shown in the centre of Figure 2 where the FoVs of two sensors are actively being angled at the rail tracks ahead. As especially the FoV of the 1D-LiDAR is severely limited, it is crucial to be able to control the exact orientation of the setup in order to reliably hit targets with the laser beam, even at distances above 1000 m. The actuation system of such an active vision sensor setup is therefore the focus of this research work.

To realize this, our work consists of the development and evaluation of a novel pointing mechanism suitable to our target application. More precisely, the contributions of our development are the following:

- A novel, high precision, versatile, low-cost pointing mechanism.
- An evaluation of the pointing resolution and accuracy of the developed system, based on extensive indoor tests in a controlled environment using optical equipment, as well as a direct comparison to commercially available gimbals.
- An investigation into the remaining sources of errors, a thorough evaluation of their effects, and proposals for their future mitigation.
- A novel proposal for an integrated sensor solution to Long-Range Obstacle Detection, consisting of custom high-precision hardware and specialized sparse long-range sensors.

The developed pointing mechanism could potentially be used for a variety of applications, ranging from active vision when combined with a camera, such as used in surveillance, astronomy, or cinematography, through measurement tasks, when combined with or Laser or Radar for example for Geomatics, or for communication systems where they orient antennas or lasers for long-range data transmission.

For our target application of Long-Range Obstacle Detection for railway systems, it is important to reliably detect and localize objects at greater distance (>1 km) requiring an orienting system with sufficient precision and accuracy to hit a target using the 1D-LiDAR at these ranges. The major requirement to our developed system is therefore the pointing accuracy and precision. Precision here refers to the spread of the orientation error when aiming at a specific target. Accuracy on the other hand refers to how true the observed orientation of the gimbal is compared to its actual orientation. The average human width at shoulder height (bideltoid shoulder breadth) worldwide exceeds 400 mm for both men and women [6]. A pointing accuracy of ±20cm at our target range of 1500 m should therefore be a minimum requirement. This translates to a maximum angular accuracy of 7.639 mdeg, rounded down to 7.5 mdeg.

Further technical requirements to the developed system are a range of motion of at least 60 deg to cover the train’s FoV as well as a payload capability of 2 kg. An additional requirement to the overall project of developing this device is the low target price. This is limited to $1000, necessitating the development of a low-cost solution, utilizing mostly common and cheaply available components.

The remainder of the paper is organized as follows: In Section 2, an extensive summary of related work in LROD and high-precision pointing mechanisms is provided. Furthermore, in Section 3, our system for precise pointing is described in detail. Experiments, results, evaluation, and a discussion thereof are given in Section 4 and a brief cost analysis in Section 5. Finally, Section 6 provides a conclusion with an outlook on future work.

## 2. Related Work

### 2.1. Railway Obstacle Detection

Long-Range Obstacle Detection for railway applications is an ongoing challenge and the focus of much ongoing research. Many approaches for purely vision-based object detection in the railway domain have been developed [7,8,9,10,11,12,13,14,15,16,17,18,19,20,21]. These typically employ machine learning and computer vision methods for object or anomaly detection or 3D-Vision methods for detecting physical intrusions into the vehicle’s path. However, most of these do not provide any in depth information. Some approaches employ machine learning or data-driven methods for depth estimation with reasonable success [22,23], even for distances over 500 m [24], but this is very much dependent on training data. Another approach is to deduce the distance based on the parallax of the tracks [25,26]. Ristić-Durrant et al. [3] provide an in-depth analysis of purely optical-based object detection systems for railways and compare their performances and limitations. Simple stereo camera setups can provide dense and accurate depth information, but due to the limited baseline only at a short range of approximately 80 m [4,27,28,29]

Ukai [30] employs an active vision approach to extend the range of vision-based approaches. In further research, this is fused with Radar sensors for depth information [31,32]. However, the setup only has a depth estimation range of 200 m with a resolution of over 2.5 m on top of the limitations of Radar regarding target materials, limiting its usability for high-speed railway applications. Similarly, Radar is also fused with fixed cameras or stereo cameras [33].

Several approaches employ thermal cameras as an easy means of detecting a variety of objects in the vicinity of the railway tracks [34,35]. These are especially beneficial in low-light situations or for detecting humans or animals due to their body heat. However, these approaches fail to detect obstacles that have the same temperature as their environment (e.g., fallen trees) and are also unable to provide any depth information. A combination of RGB and thermal cameras can provide more reliable object detections, especially in low light situations, but obtaining range information is still difficult, even when employing machine learning approaches [36].

Ristić-Durrant et al. [37,38,39,40] have developed an advanced sensor setup, by combining a multi-baseline stereo camera setup with a thermal camera and a dense LiDAR scanner to detect objects on the tracks at ranges approaching the 1000 m mark. Fusing a simple camera and a LiDAR scanner [41,42,43,44,45] has shown promising results at ranges between 50 m and 300 m, depending on object sizes.

### 2.2. Long-Range Obstacle Detection

LROD has also been in development outside the railway industry. For example, it is used in the field of remote sensing, where simple vision-based object detection can be performed at a long range through the use of super-resolution cameras or super-resolution post-processing using deep learning [46]. In this case, challenges arise from the processing of the large, high-resolution images in a timely manner [47,48,49,50].

Prior knowledge about the kind of objects that could be present in the camera’s field of view can significantly improve the long-range detection process, for example, for airplanes on a blue sky [51] or vehicles [52] on land.

Due to their dense depth maps and comparatively cheap hardware setup, stereo cameras are one of the most commonly used methods for tracking objects and their depth. In several setups, ranges between 150 m [53] and 300 m [54,55,56,57] have been be achieved if the camera baseline is sufficiently large. Deep-learning based stereo vision approaches can improve the depth estimation results but are limited in increasing the maximum range [58,59,60].

Detecting far-away objects in LiDAR is challenging, as the point clouds become sparse at long ranges and do not provide a sufficient amount of information to be able to distinguish individual objects.

Fusing camera data with other sensor modalities can equally improve the LROD performance, but this is still limited to the range of either sensor. Various combinations have been tested, such as combining Stereo Vision and LiDAR [61,62] or Radar [63]. Special long-range LiDAR sensors are in development, but still, only rarely reach distances beyond 200 m [5].

1D-LiDAR sensors can measure over much greater distances [64], reaching multiple kilometers. These sensors, however, do not provide enough data to be able to detect objects on their own and therefore need to be intelligently fused with other modalities and orienting mechanisms to provide satisfactory results.

### 2.3. Pointing Mechanisms

The various uses of pointing mechanisms and their specific requirements have led to the development of a multitude of possible orienting device designs and the respective selection of mechanisms, actuators, and sensors. The multitude of gimbal designs can be classified according to their mechanical setups, their type of actuators, their degrees of freedom or whether they orient the actual sensor, or just the optical path of the sensor setup, for example, through a mirror [65]. Most commonly, orienting devices are classified according to their mechanical setup into serial, parallel, or spherical mechanisms, as described in the following section.

**Figure 3:**
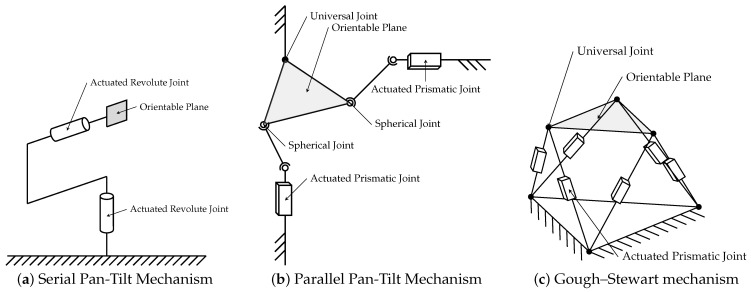
Kinematic visualization of the most common pointing mechanism designs consisting either of (**a**) serial or (**b**,**c**) parallel mechanisms.

**Serial Mechanisms:** The most common design in commercial gimballing systems consists of a serial mechanism, which places rotary actuators at the joints, typically on orthogonal axes, to achieve the largest possible workspace, as seen in Figure 3a. The disadvantage of this design is a limited accuracy, as weight and therefore the load on individual joints adds up from stage to stage and errors accumulate. Approaches to creating high-precision serial gimbals are the utilization of high-precision components, such as actuators, bearings, and encoders, materials, and manufacturing techniques. This can result in high pointing accuracy, but also high manufacturing costs [66,67,68,69]. A common problem in such mechanical apertures is the backlash, which occurs when there are small gaps or clearances between mechanical parts, which lead to a loss of motion or no transfer of force from one part to the next. This, for example, is introduced, for example, by reduction gears [70], but can be compensated using springs [71] or flexure hinges [72]. A further mechanical analysis of joint clearance influences is done by Bai et al. [73]. However, these solutions typically result in more expensive manufacturing and higher required actuation forces.

Instead of placing sensors directly on the orientation platform, mirrors can be placed there to only adjust the optical path of the sensors [74,75]. While this lowers required payload capacities and may allow for a larger range of motion, it introduces difficulties in controlling the system and promotes errors through sensor and axis misalignment [76] which need to be compensated for. Additionally, some sensor modalities, such as certain laser based devices, do not work using a mirror. Furthermore, different ways of actuation have been proposed either to save weight or to perform in particular environments, such as underwater. The use of pull-cables to actuate the different gimbal axis has been proposed multiple times [77,78], as well as the use of piezo-actuators for single Degrees of Freedom (DoF) gimbals [79]. However, the typical setup of two or three orthogonal axes has several shortcomings, such as singularities, gimbal-lock [80], and limited space for sensors, whose special designs aim to overcome, for example, through the use of more joints at non-orthogonal angles [81]. These solutions in turn have their own limitations, such as more mechanical joints, which due to their individual clearances may result in larger accumulated errors.

**Parallel Mechanisms:** parallel variants have been developed in response to the numerous shortcomings of serial pointing mechanisms. In these, several joints and actuators are placed in parallel, resulting in more complex kinematics and control methods, but also the possibility to design specific dynamic capabilities of the system. One example of parallel mechanisms is the OmniWrist III [82], whose kinematics and control have been the focus of extensive research [83,84,85,86,87]. As the proposed application is a laser communication system [88], it has also been proposed to use Bragg Cells for precise beam steering [89,90,91], in combination with the OmniWrist III, to achieve high-precision and a high range of motion [92]. Another variation of these mechanisms places the sensor setup on a universal joint and attaches linear actuators to it either in the form of push rods [93,94,95,96,97] (Figure 3b), pulleys [98,99] or pneumatic muscles [100] to orient the platform.

Flexural hinges, which connect rotary motors to the gimbal platform, can also provide beneficial dynamic properties in such a setup [101], while flexure hinges instead of a universal joint greatly benefit the system’s accuracy by limiting backlash [102,103]. Such a setup can also be extended to stereo vision, where both cameras need to be moved parallel to each other around their centers [104]. Gough–Stewart platforms (Figure 3c) and variations thereof feature more than the two or three DoFs required for a typical pointing mechanism but are equally often used for this purpose [105,106,107,108,109,110,111]. Piezo-actuators in the legs of this type of platform can provide high accuracy, but only a limited range of motion. The combination of multiple actuators in each leg can incorporate the advantages of both [112]. Furthermore, different leg designs for this platform approach, using a variety of actuators and featuring different kinematic characteristics [113,114,115,116,117,118,119,120,121], have been proposed, but few have actually been manufactured and tested [122]. Another possible improvement to the Gough–Stewart platform is the use of flexure hinges to reduce backlash and nonlinear friction [123]. Placing additional constraints on the system, for example, by fixing one of the joints, has also been suggested as a way to improve the controllability of the system [124].

A special set of tip-tilt parallel mechanisms has been developed for the use in satellites, where a high-precision but low range of motion is required. These typically employ flexure hinges, as these provide no friction, backlash, or wear, and a high-accuracy actuation part, such as piezo-actuators [125,126,127], electromagnets [128,129,130] or servos [131]. However, the use of such flexure hinges can also lead to problems such as the distortion of the mirror surface at larger deflections [132]. Using a flexure ring instead of individual hinges attached to the mirror can compensate for this [133].

Further variations on parallel gimbal platforms using various actuating arm designs include the use of three rotating legs to simulate the three DoF motion of human eyes [134], and installing flexure hinges on the actuating legs used to carry the large load of a rocket engine [135]. For underwater usage, it is beneficial to have all actuators in a single physical location to simplify the water-proofing process. To solve this issue, a specific 3DoF gimbal has been developed [136], featuring pan, tilt, and zoom motions, or similarly featuring only 2DoF [137]. Additionally, several more theoretical designs, which have not been tested, have been proposed and their dynamics analyzed [138,139,140,141,142].

Parallel gimbal mechanisms are also often used in Micro-Electromechanical Systems (MEMS) applications [143] to tilt mirrors found in image projectors and LiDAR devices, though again with a very limited range of motion.

Parallel gimbal designs offer the possibility of designing complex kinematic mechanisms for special target applications. However, these designs typically require a larger number of joints and their respective possibilities for backlash and special, expensive actuators, such as piezoelectric actuators or linear motors. Furthermore, their kinematics cannot be easily derived, making precise control mechanismsand active stabilization more complicated.

**Spherical Mechanisms:** A niche group of pointing mechanisms consists of spherical mechanisms. For the use as an animatronic eye, spherical cameras can be suspended in a fluid and be actuated using electromagnets [144]. This allows a highly dynamic motion but does not feature precise pointing control. Special motor designs with multiple DoFs can also be used as spherical actuators. This can be achieved through special rotor/stator designs [145,146,147,148] or ultrasonic motors [149] but limits the available payload capacity, space for sensors and overall range of motion of the system.

Even though a large variety of gimbal designs exists, most inaccuracies stem from the same sources. Fisk et al. [150] analyze possible sources of error, such as runout of bearings, non-orthogonality of axes, or the misalignment of other positioning sensors. Sweeney et al. [151] provide a set of general design rules for high-precision pointing mechanisms. These focus though on classical serial and parallel mechanisms and mainly focus on utilizing expensive and high-quality components.

The gimbal developed as part of our research can be assigned to the category of serial gimbals, as one actuation stage is based on the other. Through the use of reduction gears and lever mechanisms, the actuator and encoder resolution can be down-stepped significantly, increasing both the system’s accuracy and precision. The separate mounting points for the payload carrying joints, independent of the actuators, partially isolate influences of backlash onto the orienting platform.

A detailed overview of proposed gimballing mechanisms is given in Table 1. This table shows both commercially available orienting mechanisms and proposed mechanisms from the available literature.

## 3. Methods

In this section, the developed design is introduced. First, we explain the general kinematic setup, consisting of an XY-Table and a double gimballed sliding lever setup. We then elaborate on our selection of mechanical actuators and transmission mechanisms and derive the overall system kinematics. By using integrated position encoders and custom control electronics, the system can be controlled in a closed-loop manner.

### 3.1. Proposed Orienting Mechanism

**Figure 4:**
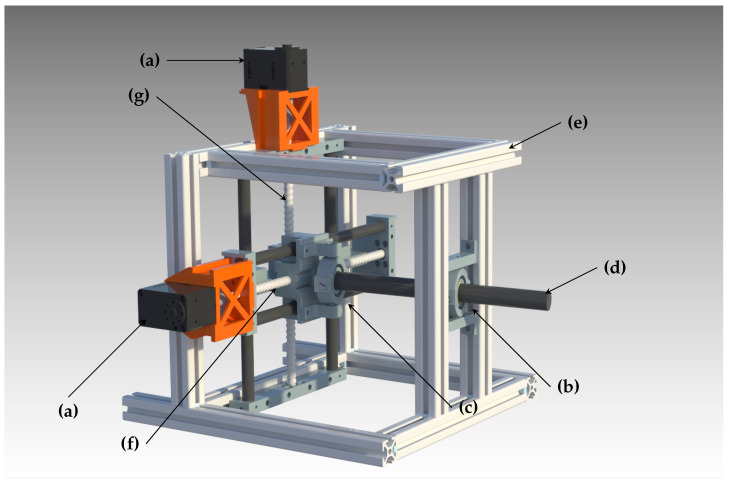
Full system CAD: (**a**) Dynamixel servo motors; (**b**) frontal universal joint with linear sliding bearing; (**c**) second universal joint; (**d**) central lever shaft and sensor mounting point; (**e**) system frame; (**f**) XY-Table pan axis and ball screw; (**g**) XY-Table tilt axis and ball screw.

The Computer-Aided Design (CAD) of the most relevant sections of our gimbal is depicted in Figure 4. The central part of our proposed mechanism is the double gimbal lever system, which transforms a 2D translation from our actuators into a full 3D rotation. A solid shaft acts as the major orientation component at the core of this design, rotating around the center gimbal/universal joint while being actuated by the other one. The strength and extension of the rod give few limitations to the available space and carryable weight. Consequently, various sensors can be mounted at the end of the shaft with no interference with the gimbal frame.

The lever not only performs the task of acting as the rotation platform but also serves the function of a mechanical reduction, reducing a longer low-torque motion into a shorter high-torque motion, thus decreasing the actuated step size and increasing the system’s precision.

The actuation of the proposed double gimbal lever system is performed using the XY-Table, a part commonly found in 3D printers or Computer Numerical Control (CNC) machines. The mechanism consists of two orthogonal linear actuators that translate a working plane along two separate axes. Most commonly, for CNC machines, a workpiece would be mounted to this plane and passed through a mechanical processing procedure, such as a mill or lathe. The widespread use of this mechanism makes it possible to easily obtain high-quality and low-cost price components to assemble this subsystem and integrate it into our design.

The central lever and the XY-Table are combined using the second universal joint, through which the lever endpoint is attached to the actuation plane of the motorized table. As a consequence of this arrangement, one endpoint of the central shaft is always located in the actuation plane, and the rotation point of the lever is locked in the platform frame using the front gimbal, resulting in a rotation motion around the latter. To adjust for the distance variation between the two gimbals, the former includes a sliding bearing that enables the central lever to move inwards and outwards. Additionally, this means that the sensor setup does not perform a pure rotation, but a translation along the pointing axis (a screw motion in total), which can be computed and accounted for in software, and does not significantly impact long-range optical sensing.

**Figure 5:**
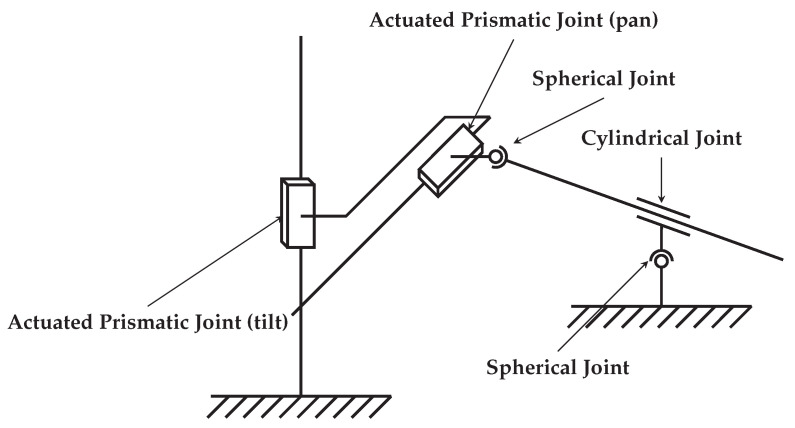
Kinematic visualization of the proposed high-precision pointing mechanism. The two actuated prismatic joints comprise the XY-table, while the two spherical and the cylindrical joints make up the lever sub-system.

A complete kinematic diagram of the mechanism, highlighting the two prismatic joints that form the XY-Table as well as two universal joints for the lever system, is shown in Figure 5.

### 3.2. Actuation and Transmission Mechanism

The kinematic chain that actuates the final sensor mounting platform through the center shaft starts with two simple, commonly available rotary motors. The aim is to precisely control the position over the XY-Table. For this, a conversion from rotary motion to linear is required. An efficient way to achieve this is by using a linear spindle coupled with a ball screw bearing. This solution is widely used in the linear precision industry, for instance, amongst CNC machine makers, where it is generally required to have higher torques and smaller displacements. A spindle is fundamentally characterized by its pitch distance *l*, which is essentially the displacement of a ball screw after one spindle rotation. The rotation angle of the spindle Δθ and linear movement of the ball screw Δx can consequently be linked using the cross-multiplication:

(1) $$\Delta x=\frac{l}{2\pi }\Delta \theta$$

For precision mechanisms, ball screws can be pre-loaded to reduce clearances, backlash and improve accuracy. This is typically done by manufacturing the ball bearing slightly smaller than the balls themselves and press-fitting them into the case. The torque required for movements is increased through this process, but backlash can be reduced to virtually zero. Stabilizing and fixing the moving platform of the XY-Table is done using linear guides and linear polymer bearings for a low-cost but stable and low clearance mounting solution.

As previously described, the transmission mechanism consists of a double gimballed lever system. Placed after the XY-Table, the lever represents an essential system component. With the main task to convert linear motion back to rotary, this element also defines controllability, stability, and overall pointing speed. The lever’s length sets the balance between pointing precision and range of motion. By increasing this parameter, precision increases at the expense of decreasing pointing speed and increasing the frame size.

**Figure 6:**
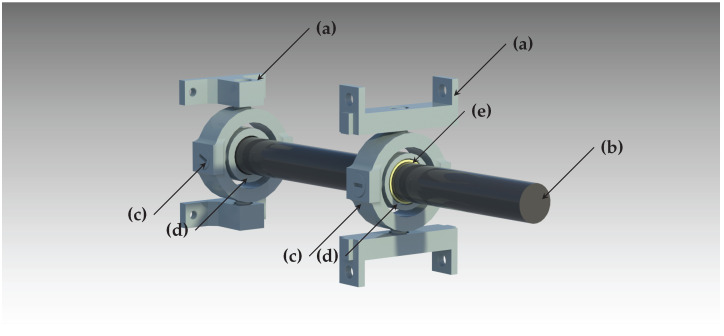
Double-gimbal lever system for converting a translatory motion into a rotary one: (**a**) gimbal mounts; (**b**) central orienting shaft; (**c**) outer segment of universal joint for pan motion; (**d**) inner segment of universal joint for tilt motion; (**e**) circular joint in the form of a linear sliding bearing.

The central shaft is mounted using two universal joints, which act as gimbals to enable the rod’s pitch and yaw rotation. These joints are realized using 2 round concentric cases, mounted using orthogonally placed ball bearings, permitting the rotation around two axes. Similar pre-loading as described earlier is done through press-fitting to reduce backlashes. This is done equally for both universal joints (see Figure 6).

As one universal joint is static, while the other one moves within the actuation plane of the XY-Table, the distance between the two of them varies. A sliding joint is used to provide this extra degree of freedom required while enabling the shaft to freely move through the frontal universal joint. This is implemented using another linear polymer bearing to minimize clearances and backlash introduced into the system.

### 3.3. Actuator Selection

High-precision actuators can significantly add to a project’s cost, especially when factoring motor drivers, higher-level controllers, and communication interfaces. By using a completely integrated package, great results can be achieved at a low price point. After testing out some options, the system’s motor choice fell to the Dynamixel XL430-W250-T [161].

This motor consists of a completely integrated package, combining a Direct Current (DC)-motor, gearbox, driver, and encoder. Positioning, speed, and acceleration are controlled using a Proportional–Integral–Derivative (PID) closed-loop system that relies on a 4096 step encoder for feedback. Furthermore, the package includes a 258 to 1 reduction gearbox that is placed in-between the DC-motor and position encoder to identify and control any potential backlash.

Several package functions are based on an integrated microcontroller, which also enables a network interface to the outside to provide control commands via a half-duplex asynchronous serial connection. The final connection to a computer for the control interface is achieved using a custom-built Universal Serial Bus (USB) to half-duplex serial interface, which also integrates the power supply unit for the motors. The power supply consists of a simple commercially available buck converter, which reduces any DC input voltage to the required 12 V. Additionally, the manufacturer provides a Software Development Kit (SDK) to interface with the serial connection of the motors via the pre-defined protocol. This SDK is both available for Robot Operating System (ROS) [162], and Arduino to integrate further high-level real-time controls.

Significant characteristics of the selected actuator for the represented use case are stall torque and encoder resolution. The Dynamixel’s built-in 4096 steps/revolution encoder provides the system with the needed resolution for this application. Furthermore, as precision mechanisms in general, and especially ours, use several press-fittings to reduce joint clearances and backlash, a non-negligible actuation force is required, even through the several reduction gears. The maximum of 1.5 Nm of stall torque can apply a sufficient force to also overcome static friction, even for small and slow movements. Further specifications of the selected actuator can be seen in Table 2.

Using 3D-printed mounts, these motors are directly attached to the mechanism’s overall frame. Custom milled motor fixations attach the motor output directly to the corresponding ball screw without any further gears.

An alternative to using the fully integrated package would be a custom built solution. Significantly higher resolutions, torques, and speeds could be achieved as well using high-quality DC motors combined with harmonic drives [163], which exploit the deformation of gears to achieve high reduction rates and low backlash, as well as high-precision encoders [164]. Even individual components of such a setup will have significantly higher costs than the fully integrated package used for this research, and were therefore deemed over the price limit. A fully integrated motor setup using such high-quality components is the ANYdrive motor [165], but this was again above the target price point.

### 3.4. System Kinematics

**Figure 7:**
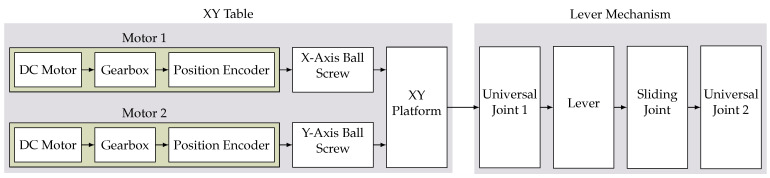
Block diagram visualization of the kinematic chain of the proposed mechanism, highlighting mechanical components inside the servo motors as well as the two subsystems, the XY-Table as well as the lever mechanism.

A further in detail description of the kinematic transmission of movement from the integrated DC-motors to the final actuated sensor platform can be seen in Figure 7. According to this serial chain and by separating the individual axes of the mechanism, we can perform a simple analysis of the theoretical resolution and precision of the system, as well as its future movement control.

Due to the integrated position encoders and the respective PID controllers, both motors can be interpreted as complete and independent subsystems. Their outputs are the respective rotary motor positions $\theta_{1}$ and $\theta_{2}$. Using the ball screw lead, which is identical for both ball screws, the *x* and *y* positions of the XY-Table can be computed as

(2) $$\begin{array}{cc} x & =l\times \theta_{1} \end{array}$$

(3) $$\begin{array}{cc} y & =l\times \theta_{2}, \end{array}$$

on which the first universal joint is placed. The conversion of this linear movement into a rotary pitch (ϕ) and yaw (ψ) is performed by the lever in combination with those two universal joints, as shown in Figure 6. The system’s attitude can then be computed as

(4) $$\begin{array}{cc} \psi & =\arctan(\frac{x-x_{c}}{D}) \end{array}$$

(5) $$\begin{array}{cc} \phi & =\arctan(\frac{y-y_{c}}{D}), \end{array}$$

where ($x_{c}$, $y_{c}$) are the center’s coordinates of the XY-Table, and *D* the separation of the two universal joints when the lever is not deflected at all. From this, the theoretical pointing resolution

(6) $$\begin{array}{cc} \Delta \psi & =\arctan\left(\frac{l\times \Delta \theta_{1}}{D}\right)=\arctan\left(\frac{4\times 360}{360\times 4096\times 100}\right)=5.59\times 10^{-4}=0.559\;\mathrm{mdeg} \end{array}$$

(7) $$\begin{array}{cc} \Delta \phi & =\arctan\left(\frac{l\times \Delta \theta_{2}}{D}\right)=\arctan\left(\frac{4\times 360}{360\times 4096\times 100}\right)=5.59\times 10^{-4}=0.559\;\mathrm{mdeg}, \end{array}$$

based on the reported Dynamixel motor resolution $\Delta \theta =\frac{360}{4096}$ = 0.08789${}^{\circ }$/step, the ball screw lead *l* = 5 mm/rev and the lever length D=100mm can be computed. This accuracy computation is an approximation, assuming the system operates close to its origin. As the system moves further away from the center point, out of the region of linear approximation of the arctan function, the resolution will increase, resulting in smaller and smaller angular step sizes in comparison to the respective motor steps.

The mathematical approximation of the gimbal model enables an understanding of the impact of design changes and component selection on the overall system performance. It can be observed that the pointing resolution is proportional to the motor resolution

(8) $$\begin{array}{c} \Delta \psi \propto \Delta \theta . \end{array}$$

The pointing resolution is proportional to the ball screw lead

(9) $$\begin{array}{c} \Delta \psi \propto l. \end{array}$$

In addition, the pointing resolution is inversely proportional to the separation of the rotation center to the XY-Table plane

(10) $$\begin{array}{c} \Delta \psi \propto \frac{1}{D}. \end{array}$$

By further analyzing this chain of mechanical components, we can derive a generic kinematic description of the system for classifying it according to its joint placement. The XY-Table consists of two prismatic joints in series, making it a PP-mechanism. The lever mechanism is held in place by two universal joints as well as a cylindrical one to enable the sliding motion. This constitutes a UCU-mechanism. As both parts are placed in series, this results in a PPUCU-mechanism. Some notations place a line above the actuated joints, making this a $\bar{P}\bar{P}\mathrm{UCU}$ mechanism.

### 3.5. Clearance and Error Analysis

Even though our design provides a great down stepping of the original motor’s resolution, the kinematic chain in Figure 7 also highlights many joints and mechanical interactions, which introduce the possibilities of clearances, backlash, bending, and other possible sources of error. The following section highlights an analysis of possible weaknesses of the mechanisms and how much they could potentially impair the system’s precision. An overview is also given in Table 3. Furthermore, we demonstrate approaches that were taken to mitigate possible issues.

#### 3.5.1. Motors

The utilized Dynamixel motors come with an integrated gearbox. As stated in Section 2, georboxes are a frequent source of possible backlash. Some advantages of using the Dynamixel motors are, on the one hand, the precise industrial manufacturing, which already results in very low tolerances in the mechanism, and on the other hand, the backlash compensation happening while controlling the motor’s position due to the rotary encoder being placed at the end of the motor’s kinematic chain.

#### 3.5.2. System Kinematics

**Figure 8:**
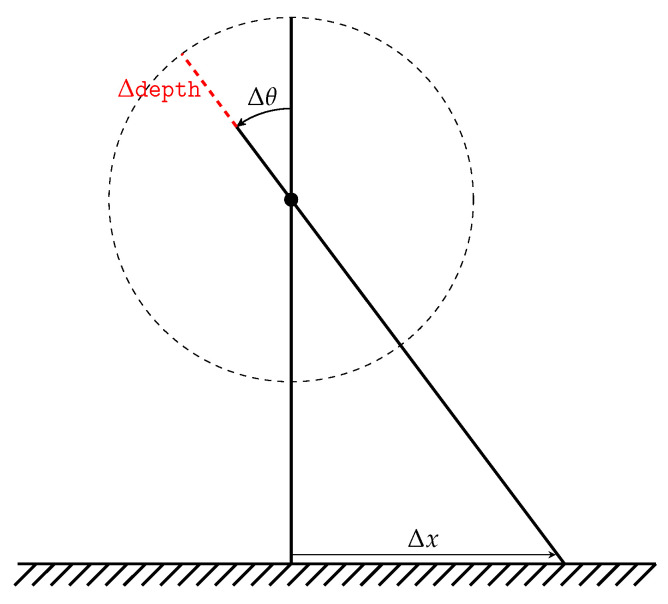
Translation error introduced through rotation with a fixed-length shaft.

The kinematics themselves do not result in a pure rotation, but there is also a falsely introduced translation of the sensor platform. Based on the system geometry shown in Figure 8, this can be calculated to be

(11) $$\begin{array}{cc} \Delta \mathrm{depth} & =d_{g2g}\left(\psi_{max}\right)-d_{g2g}\left(\psi_{O}\right) \end{array}$$

(12) $$\begin{array}{cc} \Delta \mathrm{depth} & =\frac{D}{\cos(30\;\deg)}-\frac{D}{\cos(0\;\deg)} \end{array}$$

(13) $$\begin{array}{cc} \Delta \mathrm{depth} & =15.47\;\mathrm{mm}, \end{array}$$

using the gimbal to gimbal distance $d_{g2g}\left(\psi \right)$, based on the system’s deflection angles $\psi_{max}=30\;\deg$ and $\psi_{O}=0\;\deg$:

For the target application of LROD, this is not only a negligible deviation but also a predictable one and can therefore be disregarded for further development.

#### 3.5.3. Ball Screws

Ball screws are an excellent option for precision mechanisms, as they can already come pre-loaded by their balls through the respective ball assembly [163]. The advantage of these is very low friction at high torque capabilities, which makes them a common, well-established, and affordable solution. The high-torque capability is especially beneficial due to the resistance created from the pre-loading of bearings within the various components. Sweeney et al. [151] also discusses the utility of ball screws in precision mechanisms, and how these can be utilized effectively.

Ball screws can introduce two possible kinds of play into the mechanism: axial and radial. In our case, radial play is immediately compensated through linear guides mounted parallel to the ball screws. Axial play, however, can affect the accuracy of the system and the manufacturer’s pre-loading of the ball assembly is therefore of high importance [166].

Unfortunately, as the utilized ball bearings were already available in our workshop, no manufacturer information is available about them. There does not seem to be any observable axial play, but the pressure exerted from linear guides on either side can also provide some pre-loading to reduce clearances, should there be any.

#### 3.5.4. Linear Guides

According to the manufacturer’s specifications [167,168], there is a radial fitting tolerance of $\epsilon =10^{-2}\;\mathrm{mm}$ between the guide shaft and the respective shaft of the linear sliding bearing. A direction change of one axes could result in a backlash in the magnitude of the fitting tolerance, which cannot be ignored. Assuming all other variables remain constant, this could, according to Equation (6), result in a worst-case angular inaccuracy of

(14) $$\begin{array}{cc} \Delta \psi & =\arctan\left(\frac{\epsilon }{D}\right)=\arctan\left(\frac{10^{-2}}{100}\right)=5.729\times 10^{-3}=5.729\;\mathrm{mdeg}. \end{array}$$

This is, however, compensated by several effects:

- Press-fitting the bearings into the respective structural part will result in a more optimal, tighter fit.
- As two linear guides are used in parallel with a very tight fit, both bearings will be pre-loaded thus reducing any possible play [169].
- In addition to the two linear bearings for each axis, there is also the ball screw, fixating the moving platform further and reducing possible backlash.
- By controlling the movement protocol of the XY table, it can be assured that all bearings are always on the same side of their clearances during a backlash and therefore compensating further for possible backlash.

#### 3.5.5. Universal Joints/Gimbals/Ball Bearings

Ball bearings are utilized at various locations throughout the mechanism, especially inside the two universal joints that fixate the orienting lever. Axial play of the balls in these locations could dramatically affect the overall pointing precision of the device. During a simple direction change of motion, this clearance could cause a backlash in both universal joints, having double the effect on the pointing accuracy. Our design utilizes ball bearings by SKF featuring a ϵ=7µm axial play [170,171]. A deviation of 7 µm in both gimbals could lead according to Equation (6) to an error of

(15) $$\begin{array}{cc} \Delta \psi & =\arctan\left(\frac{2\times \epsilon }{D}\right)=\arctan\left(\frac{2\times 7\times 10^{-3}}{100}\right)=8.021\times 10^{-3}=8.021\;\mathrm{mdeg}. \end{array}$$

Again, several compensating effects are utilized:

- The most significant compensation action is the pre-loading of any bearing. This can be done in two ways: Simple press-fitting of the bearing onto the shaft and inside the casing will apply a significant force onto it and thus will permanently keep it at one side of the bearing’s clearance and reduce backlash [163].
- Spring-loading is a second method to reduce a bearing’s backlash but is not utilized in our design.
- Finally, again some backlash can be compensated for in the controls and calibration of the mechanism, though this can be unpredictable and might suffer in dynamic environments with high vibrations.

#### 3.5.6. Frontal Linear Guide

The rotation center’s linear guide provides the extra degree of freedom for the system to transform the XY-Table translation into a rotation freely. The main shaft only moves inwards and does not conduct any rotary movement with respect to the linear bearing. The sleeve was purposely lengthened in this location to 20 mm to reduce the effect of any potential radial play. Low clearance and proper compatibility are especially ensured by having both the shaft and its sleeve manufactured by the same company. Furthermore, payload mass and gravity as a restoring force provide a degree of pre-loading onto the bearing, but further measures to reduce possible clearances could be applied in the future.

#### 3.5.7. Bending Moments

The bending of the central shaft or other mounting components could introduce errors to the pointing accuracy. However, most components are milled out of aluminum and significantly over-dimensioned for the expected future payload. Furthermore, it is also assumed that should there be any significant bending effects; these should be constant in static measurement situations and would therefore only introduce a linear offset which can be compensated for in calibration.

#### 3.5.8. Symmetry

Symmetric designs can compensate for many sources of errors like thermal expansion or bending in high-precision mechanisms [172]. As the linear stages, gimbals and chassis are fairly symmetric, the design benefits of symmetry error advantages. The book highlights the good practice of placing measurement axis in symmetry planes, balancing the effects of thermal expansion affecting either side of the symmetric plane equally. This practice has been adopted on many occasions throughout the design, specifically in the linear stages of the XY-Table and gimbals for the lever mechanism, but also the overall system frame.

### 3.6. Encoder and Position Feedback

In most common pan-tilt serial gimbal mechanisms, the encoder is placed at the rotational joint providing a direct measurement of the current orientation, enabling complete closed-loop control and the capability to adjust for any backlash or other errors. For high-precision measurements, this requires a very high-resolution position encoder, such as an EAM580-B [173]. However, even these expensive encoders only feature 14-bit single turn precision resulting in an angular step resolution of 21.97mdeg.

Our proposed mechanism utilizes the cheap AS5601 12-bit encoders [174] which are integrated into the motor package. As these are placed earlier in the mechanism in front of several down-stepping mechanisms, we can utilize the encoder over multiple motor turns, achieving a much higher step resolution, resulting in a final angular resolution of 0.559mdeg. The disadvantage of this placement is that the encoder is not at the end of the kinematic chain (as seen in Figure 7). This means that parts of the mechanism do not provide position feedback and therefore run in open-loop control. In the future application, this can be extended and improved using the sensors available in the to be mounted sensor platform, such as cameras and Inertial Measurement Unit (IMUs).

### 3.7. Control Systems

The general control systems of the developed pointing mechanism are integrated into the Dynamixel motor package. These motors interface with a control computer using a simple half-serial duplex communication. On the computer side, an interface is created using ROS [175] and the Dynamixel SDK. Transformation functions and the system kinematics can then be easily modeled in ROS to convert angular commands into the respective motor and encoder positions.

Low-level movement controls are handled by the integrated Cortex-M3 microcontroller, which handles position and velocity PID control to precisely steer the gimbal.

As the communication via USB with the computer and ROS and the sending of reactive commands is not a real-time operation, we have developed a second operation mode. In this case, an additional microcontroller in the form of an Arduino Zero [176] is placed in between the computer and the motors. The Arduino is now capable of collecting information from various sensors and sending real-time commands to the motors for precise control. Furthermore, by also running VersaVIS [177] on this microcontroller, the current gimbal positions can be precisely timestamped. High-level commands can still be sent from the computer via ROS and ROSserial [178] to the Arduino, which are processed and sent on to the motors. This allows, for example, the integration of optical limit switches [179], similar to ones found in 3D printers, which can be used for precise calibration of the gimbal parameters and motor encoders. Furthermore, in combination with IMUs, the Arduino could send commands for stabilization and disturbance suppression to the motor, which a computer could not provide in real-time.

## 4. Experimental Evaluation

To evaluate the pointing accuracy of the developed system, we performed a series of experiments in a controlled environment. In order to perform a direct and fair comparison to commercially available systems, we performed the same experiments using a consumer-level cinematography gimbal.

### 4.1. Experimental Setup

#### 4.1.1. Environment

The experiments were performed in a controlled lab environment to ensure the highest possible accuracy. The pointing device was placed at one end of a long and empty corridor, with target points placed at the opposite end. To observe the precise movements of the orienting mechanism, a laser diode was mounted in place of the future sensor setup to highlight the targeted position at the end of the measuring range. Individual measurement points of the laser diode were recorded on graph paper located at the opposite end of the corridor. To minimize distortions, the paper was mounted to a flat board that was secured to the wall. The measuring distance between the target area and the pointing mechanism was measured using a Leica Disto D8, a separate construction grade laser distance meter with an accuracy of ±1mm [180]. It was measured from the center of rotation in the frontal gimbal of the pointing platform to the graph paper sheet and determined to be 46.341m.

The system was securely mounted to a table using clamps, and all moving parts and cables were securely fixed to the table to prevent any disturbances. The control computer from which an operator can send position commands was placed on a separate table to minimize the interactions with the system.

#### 4.1.2. System Setup

The mechatronic components of the pointing mechanism were controlled from a separate computer via a self-developed control board. On the software side, the servos are controlled using the Dynamixel SDK, provided by the manufacturer, as well as a custom ROS node, to send commands according to the specified experiment protocol. The low-level position control of the gimbal is performed by the Dynamixel internal micro-controller using a PID controller. The values for which were chosen to be: $K_{P}P=5$, $K_{P}I=0.0076\;s$, $K_{P}D=250\;1\;/\;s$.

The visualization of the targeted position is done using a laser diode. This is mounted instead of the sensor platform using a 3D-printed fixture. The utilized diode is a Picotronic DA650-1-5(11X60) [181] and has a focal length of 50m, with a beam diameter of 8mm and a beam divergence of 5.729mdeg. It is possible to visually determine a center of the projected laser point, but ultimately this places a lower limit on the evaluation accuracy of this experiment.

#### 4.1.3. Calibration

The pointing mechanism has a few mechanical parameters which are originally undetermined and can be obtained through a calibration procedure from a set of measurement points. The most significant parameter is the principal point of the mechanism. As the motor encoders set their origin at an arbitrary location, it is necessary to determine the encoder position of the pointing origin when no deflection takes place. As the lever motion does not linearly transform encoder steps into angular steps, this is especially significant. This means that the pointing resolution at the principal point is the lowest (the worst) and increases (improves) towards the edges. This nonlinearity can be used in combination with a set of measurements to determine the principal point relative to the origin of the motor encoders.

Further system calibration parameters stem from various manufacturing inaccuracies. It can be observed, that the axes of the XY-Table are not perfectly perpendicular, but slightly skewed. This defect can be determined through calibration and, therefore, can be compensated for in the experimental setup. A deviation of 0.175 deg has been observed.

#### 4.1.4. Experimental Protocol

The limited space of the long corridor does not permit a high-accuracy evaluation of the system’s full range of motion. The first step of the experiment was, therefore, to determine the maximally available range of motion, which will still result in all projected laser points lying within the target area. This range of motion was determined to be ψ∈{6.0deg,7.7deg} in pan and ϕ∈{1.0deg,2.2deg} in tilt. To prevent a human bias in the selection of target points, these were sampled uniformly within the previously determined range.

Furthermore, a control strategy has been implemented to minimize backlash and bending effects. This strategy can shortly be summarized as:

1. Input of new target position.
2. Orienting mechanism is actuated along the tilt axis to 500 encoder steps on top of the desired target tilt coordinate.
3. Orienting mechanism is actuated along the tilt axis to the desired target tilt coordinate.
4. Orienting mechanism is actuated along the pan axis to 500 encoder steps on left of the desired target pan coordinate.
5. Orienting mechanism is actuated along the pan axis to the desired target pan coordinate.

This strategy ensures that target points are always approached from the same direction (top-left), ensuring that backlash and bending effects always influence the result in the same way and can therefore be controlled.

Before the beginning of the experiment, the PID values of the integrated motor controllers were tuned manually. Convergence speed was not a priority during this experiment, so these values were chosen in order to ensure convergence with minimal overshoot.

The overall experiment protocol for recording data points was therefore:

1. Generate a new random target position.
2. Actuate the orienting mechanism to the given target position according to the previously defined control strategy.
3. On the controller side, the commanded target position is recorded, as well as the continuously reported pointing position as the mechanism acts upon the command. Additionally, the final convergence point is recorded. For later data association, each target position is assigned an ID, which is also assigned to all associated recordings.
4. The projected target point on the graph paper is manually recorded with the respective target ID.

At the end of the experiment, the data points are manually read off from the graph paper and added to the digital recording of the controller commands and values. This manual process also limits the evaluation accuracy, as points could only be read off to an approximate accuracy of 1.5mm.

### 4.2. Results

From the previously described experiment, a total number of 182 corresponding measurement points could be obtained. These lie in a measuring range of ψ∈{6.0deg,7.7deg} along the pan axis and ϕ∈{1.0deg,2.2deg} along the tilt axis. Two measurements have been removed, as they were out of the range of the manual recording process. One measurement has been removed, as it is presumed to be a manual measurement error, due to its significant misalignment, resulting in a total of 179 measurement points. The subsequent sections will analyze this data regarding the overall pointing error, and try to recognize individual sources of error within the mechanism. Manual measurement points on the graph paper have been converted to degrees based on the distance measurement to the wall of 46.341m. Recorded encoder position values have been converted to the corresponding gimbal attitude in degrees based on the previously described system kinematics and a system calibration obtained from a subset of the measurement points. The final measurement points have been aligned with the target points to remove any linear offsets in the mechanism or the experimental setup using least-squares optimization. This would also be part of a complete calibration procedure.

#### 4.2.1. Overall Pointing Error

**Figure 9:**
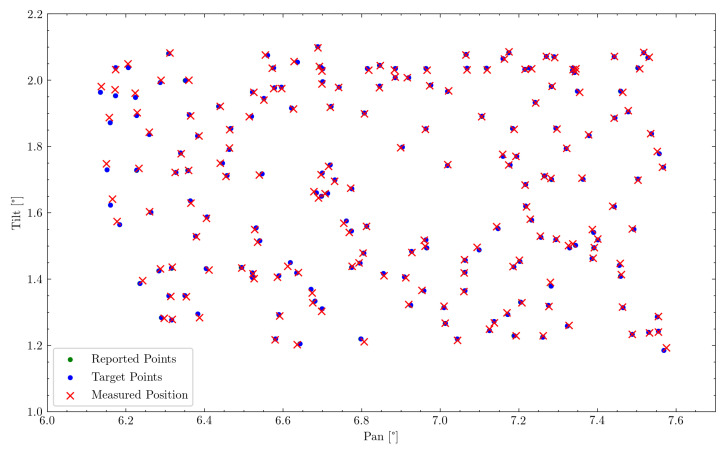
Representation of commanded (blue) and reported (green) to measured (red) pointing position. Due to good motor controls, the reported position is nearly always covered by the commanded position.

A first evaluation can be done by comparing the commanded position values to those observed in the experiment. This can be observed in Figure 9 by comparing commanded (blue) and measured (red) point positions. This can be observed to be an Root Mean Square Error (RMSE) of 6.203 mdeg, as well as a maximum error of 18.824 mdeg. Due to the motor’s internal PID-controller, the commanded position is, however, not always identical to the reached position of the motor and therefore the actual gimbal attitude. However, this error is observable through the motor encoders and therefore enables a second evaluation of the gimbal attitude reported by the encoders to the measured values. This RMSE is determined to be 6.179 mdeg with a maximum error of 18.854 mdeg, which is also shown in Table 4. In addition, 83.8% of measuring points were therefore within the required accuracy range of 7.5 mdeg.

In all further evaluations, the reported value of the motor encoders will be used to determine the relevant gimbal attitude, as this value is not subject to PID-controller tuning or other control mechanisms.

**Figure 10:**
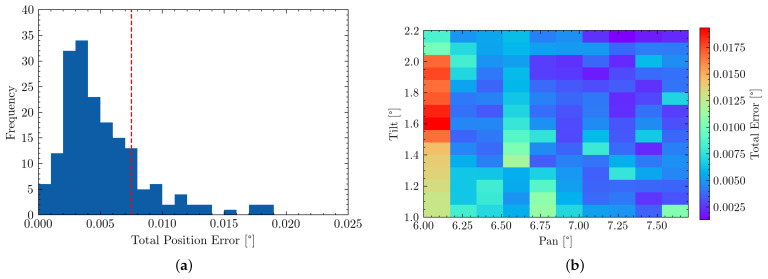
(**a**,**b**) visualize the system’s overall pointing error and its distribution. (**a**) Histrogram highlighting the distribution of RMSE pointing errors. The red line highlights our overall accuracy requirement (**b**) Heatmap visualization of pointing errors. Large red-spot due to one large mis-aligned measurement and no further points in that area.

A histogram plot visualizing the distribution of pointing errors can be seen in Figure 10a. This graph also highlights that the majority of all measurement points lie within the required pointing accuracy. The spread of pointing errors throughout the measuring range is visualized using a heatmap in Figure 10b, showing regions of larger or lower error.

#### 4.2.2. Evaluating Axis Performance

**Figure 11:**
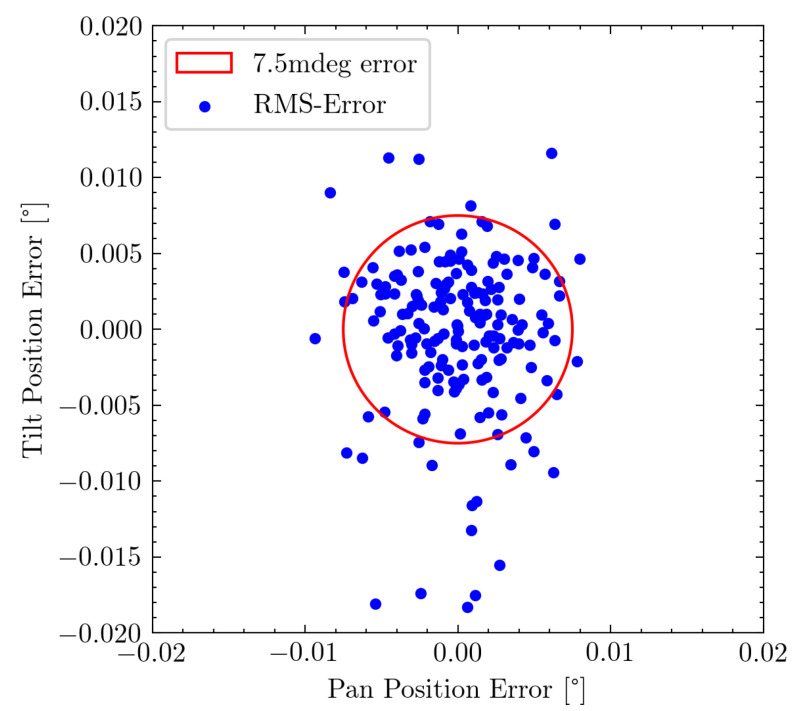
Relative RMSE of each individual measurement point. The red circle shows our requirement accuracy of 7.5 mdeg.

The pan and tilt axis of the mechanism can be regarded as separate from each other, as one does not influence the other and should therefore both be evaluated individually. Figure 11 visualizes the mis-projection of each individual measurement point. The minimum required pointing accuracy is highlighted using the red circle and shows that the majority of measurement points fulfill this requirement.

**Figure 12:**
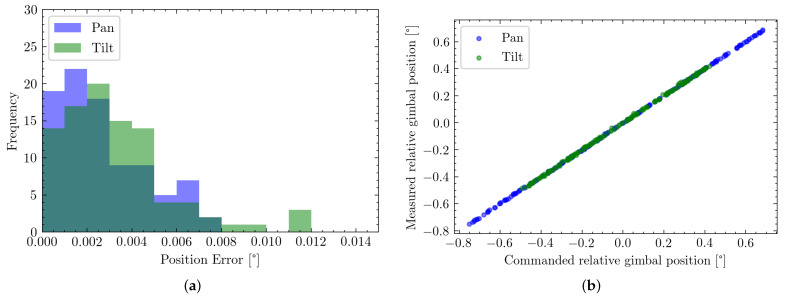
Evaluating error distribution and system linearity for both the pan (blue) and tilt (green) axis. (**a**) Histogram of error distribution along the individual axis; (**b**) Linearity of commanded position to the measured output.

Figure 12a shows the distribution of orienting errors along each axis. A slightly wider error distribution can be observed for the tilt axis. This is also represented in the RMSE values, where along the pan axis an error of 2.801 mdeg with a maximum of 9.355 mdeg is achieved, while along the tilt axis a RMSE of 3.671 mdeg with a maximum of 18.278 mdeg is observable.

Furthermore, by separating the data into individual axes, a slight skew in the mechanism is observable. This is due to a misalignment of the pan and tilt axis during assembly. A rotation of 0.175 deg of the tilt axis relative to the pan one can be determined. However, this can be compensated for in calibration and has already been included in the previously stated system calibration.

#### 4.2.3. Observing Linearity of Command and Measure Points

An essential factor, especially for later control systems, is the system’s linearity. How well do measured position and target position coincide? This is shown in Figure 12b. From both visualizations, it can be seen that the system expressed a very linear behavior. This can also be quantified by computing the correlation of each set, resulting in a value of 0.9999 for the pan axis, as well as 0.9998 for the tile axis.

#### 4.2.4. Error Source Analysis

Certain types of induced errors can be proportional to the distance travelled, the distance between two consecutive measurement points, of the mechanism. This can for example be friction or bending effects in the mechanism, which accumulate during the motion from one measurement point to the next.

**Figure 13:**
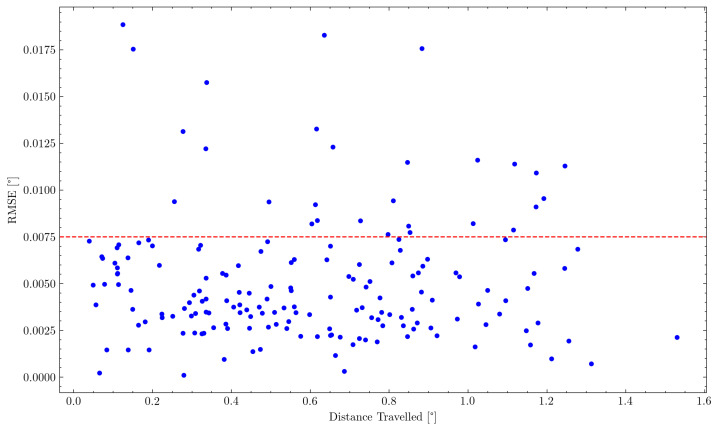
Comparison of distance travelled between two consecutive measurement points to the respective RMSE of the following measurement point.

This correlation would not have to be linear, but neither Figure 13 nor the logarithmic version revealed any distinguishable correlation.

This can mostly be accredited to the employed control methods and movement protocols, described in Section 4.1.4, which specifically aimed at reducing such errors.

#### 4.2.5. Commercial Gimbal Comparison

To perform a direct comparison to commercially available solutions in the same price segment, we repeated the same experiment using a DJI RS2 cinematography gimbal. The measurement range was determined to be 46.12 m, but due to the very limited resolution of the gimbal of only 0.1 deg, only very few measurement points could be obtained. In total, 107 points were recorded.

**Figure 14:**
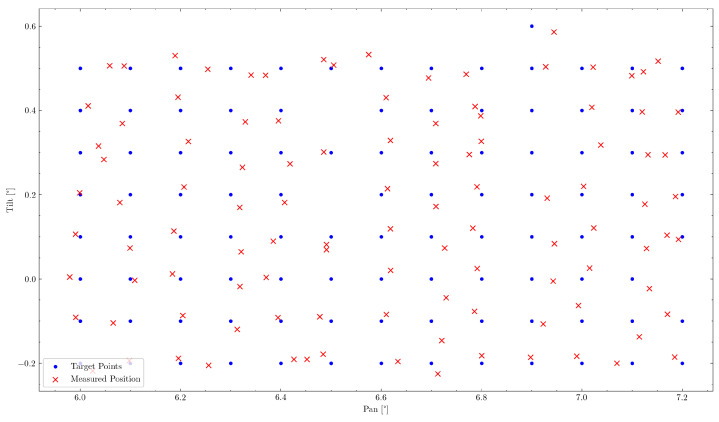
RMSE evaluation of the DJI RS2.

A similar RMSE visualiszation has been created in Figure 14 and the RMSE has been calculated to be 30.93 mdeg.

This error is significantly higher than what would be usable for our target application, but even more, the very limited actuation resolution makes this an unsuitable solution to our problem.

### 4.3. Discussion

From the reported experimental results, several observations and conclusions can be drawn regarding the system’s precision:

- The observed pointing precision is as close as possible to the evaluation precision of the experimental setup. Over the available measurement range, even deviations of single millimeters cause errors in the range of millidegrees. Therefore, the evaluation accuracy is limited by the laser-pointer’s accuracy, the marking of the experimenter, and the later digitalization of the paper markings.
  Furthermore, the recording graph paper, which was taped to a pinboard fixed on a wall, is a possible source of error by itself.
  Effectively, bending of the data recording paper might have caused the measurement distortion seen in the left part of Figure 10b. It is, therefore, hard to conclude with the available measuring equipment, whether the actual pointing accuracy is equal to the observed one or if the system is even more precise but can not be evaluated at the better precision.
- A second observable source of error comes from the linear bearing within the frontal gimbal, responsible for the free movement of the orienting shaft. This bearing has been damaged slightly during assembly, resulting in minimal play. Fortunately, performance is not significantly impacted statically due to the effect of gravity and the large diameter of both the shaft and bearing; nevertheless, the part would need to be replaced for more dynamic scenarios.
- Nonlinear bending effects in 3D printed components like the motor mounts could still impact performance. This could be evaluated using a Finite Element Method (FEM) simulation and their design could be further optimized, for example using generative design methods. Furthermore, adequate control methods could be used to compensate for these effects [182].
- As the calibration of the mechanism’s intrinsic parameters, especially the principal point, is currently done using external experiment data; these are also subject to the same experimental inaccuracies. We are therefore planning to expand the system’s controls using optical limit switches (such as ones found in CNC machines and 3D printers), to perform an automatic self-calibration.
- Furthermore, it can be observed that significantly more projection errors are observable in the tilt axis than the pan axis. This could be caused by the staging in the XY-Table, as the tilt axis has to carry more loads and might be subject to more vibrations from the series of actuators. For our future target application of Long-Range Obstacle Detection, this is not problematic, as the pan axis is more critical for covering the vehicle’s FoV.
- Oscillations during the convergence to a new measurement point due to the motor’s internal PID controls are also a possible source of inaccuracies. These can be reduced using further and more extensive tuning of the PID parameters.

## 5. Cost Analysis

One of the main goals of this project was to develop a low-cost system. The total cost of the utilized components was $1000 and is broken down in Table 5. Where components were manufactured using available in-house machinery, such as 3D printers and CNC machines, only material costs are listed.

The manufacturing cost for a comparable system using a classical serial pan/tilt design, which employs high-precision components, such as harmonic gearboxes, precision motors, encoders, and additional materials, was estimated to amount to approximately $2500.

## 6. Conclusions

In this paper, we demonstrated a high-precision pointing mechanism for future utilization in a railway Long-Range Obstacle Detection. Due to the low target price point, the system consists of simple and widely available low-cost components as well as in-house manufactured parts. By creating a novel actuation mechanism consisting of a common XY-Table and a double-hinged lever system, a very small rotation resolution was achieved. Various means of pre-loading can keep clearances in the mechanism, and therefore backlash to a minimum, resulting in very high-precision and high accuracy in the device’s pointing performance. We validated our design using a controlled indoor experiment, during which we recorded measurements over a range of nearly 50 m. Our experiment has shown that our system is capable of precisely and accurately targeting individual points at a long range, with an angular resolution comparable to that required for detecting humans at 1500 m using a long-range LiDAR. The achieved pointing precision reached what was possible to evaluate using our available experimental procedure, and highlighted the challenges of testing and calibrating high-precision equipment. For the future target application of LROD, this provides sufficient pointing precision to target and track long-range obstacles reliably. The high pan position accuracy makes this mechanism particularly suitable to explore the train’s FoV. Nevertheless, the impact of vibrations and disturbances from train operations will have to be investigated further in the future.

We plan to continue the development of this mechanism, specifically in the realm of controls, to be able to achieve high-pointing precision under dynamic scenarios, remove possible nonlinear errors from bending or backlash effects, and integrate a self-calibration procedure. Furthermore, we aim to combine this with a suitable long-range sensor setup, consisting of a 1D-LiDAR and several cameras to construct a complete Long-Range Obstacle Detection system.

## Figures and Tables

**Table 1:** Overview of existing gimbal mechanisms from commercial suppliers and research. The individual specifications are compared with our requirements to highlight that no system can fulfill them within the specified price range. For many mechanisms, exact manufacturing costs are not known. Based on the depicted components, a rough price range is estimated as $–$$$. Most systems have different ranges of motion per axis. As pan is the most relevant axis to us, in those cases, only the pan range of motion is listed.

	Resolution	Accuracy	Price	Range of Motion	DoF	Max Payload
Our requirements	7.5 mdeg	7.5 mdeg	$1k	±30deg	2	2 kg
Our solution (see Section 4)	0.559 mdeg	6.179 mdeg	$1k	±30deg	2	2 kg
DJI RS2 [152]	100 mdeg	20 mdeg	$1k	±180deg	3	4.5 kg
DJI Ronin 2 [153]	100 mdeg	20 mdeg	$8 k	±180deg	3	13.6 kg
Gremsy T3 [154]	10 mdeg	20 mdeg	$2 k	±180deg	3	1.7 kg
Standa 8MG-2SQ200 [155]	15 mdeg	n/a	$$$	±180deg	2	6 kg
Freefly Movi Pro [156]	n/a	n/a	$7 k	n/a	3	6.8 kg
Aerotech AMG200 [157]	1.39 mdeg	0.139 mdeg	$$$	±180deg	2	20 kg
Newport FG-URS [158]	0.5 mdeg	11.5 mdeg	$$	±180deg	2	10 kg
OES Pan-Tilt Stage [159]	1 mdeg	10 mdeg	$$$	±180deg	2	10 kg
FLIR PTU-5 [160]	50 mdeg	n/a	$3 k	±180deg	2	2.3 kg
Hudgens and Cawthon [66]	0.1 mdeg	5 mdeg	$$$	±67deg	2	11.3 kg
Li et al. [67]	0.0217 mdeg	0.819 mdeg	$$$	±178deg	2	5.5 kg
Ferris and Phillips [70]	24 mdeg	250 mdeg	$$$	±270deg	2	n/a
Qiu et al. [78]	n/a	3437.8 mdeg	$	±15deg	2	n/a
Rosheim and Sauter [82]	n/a	60 mdeg	$$	±90deg	2	2.3 kg
Antonello et al. [94]	n/a	0.57 mdeg	$$$	0–15 deg	2	n/a
Villgrattner et al. [93]	n/a	6.7 mdeg	$$	±30deg	3	0.04 kg
Lee et al. [101]	40 mdeg	n/a	$	±35.54deg	2	0.05 kg
Zelenika et al. [102]	n/a	n/a	$$$	±2deg	2	n/a
Huajun et al. [103]	0.0573 mdeg	10 mdeg	$$	±2deg	2	n/a
Chen et al. [107]	0.465 mdeg	n/a	$$$	n/a	6	n/a
Du et al. [125]	<5.7 mdeg	<17.2 mdeg	$$$	<± 0.026 deg	3	n/a
Burger and Eder [126]	0.389 mdeg	0.0217 mdeg	$$$	±0.017deg	2	1.3 kg
Henein et al. [127]	n/a	0.00802 mdeg	$$$	±0.001deg	2	0.075 kg
Aoki et al. [130]	n/a	0.057 mdeg	$$	±4deg	2	n/a
Geijo et al. [111]	1.39 mdeg	0.11 mdeg	$$$	±0.05deg	6	240 kg
Dong et al. [112]	<0.0057 mdeg	<0.0115 mdeg	$$$	±3deg	6	2 kg
Harvison and Hardy [131]	n/a	0.74 mdeg	$$$	±0.37deg	2	n/a
Bederson et al. [146]	11 mdeg	150 mdeg	$	n/a	2	0.015 kg
Jae-Sung Lee et al. [148]	5500 mdeg	n/a	$	±33deg	2	n/a

**Table 2:** Dynamixel XL430-W250-T specification according to the manufacturer. Of importance are especially the encoder resolution and the position control range, as well as a sufficient torque.

Characteristic	Specification
Price	$48.9
Supply Voltage	12 V
Stall Torque	1.5 Nm
Stall Current	1.4 A
No-load speed	61 rpm
Encoder resolutions	4096 steps/revolution
Temperature range	$-5-72{\;}^{\circ }C$
Dimensions (*W* × *H* × *D*)	28.5 mm × 46.5 mm × 34.0 mm
Position Control Range	±256 revolutions

**Table 3:** From the utilized components and overall system design, several sources of error can be identified and accounted for in the further steps of the system’s design.

Source of Error	Reason	Worst Error Estimate
Motor inaccuracies	Internal Gearbox	0 mdeg
Position deviation	Lever motion	15.47 mm
Linear guide	Axis fitting tolerance	5.29mdeg
Component bending	bending due to payload	5mdeg
Ball Bearing	Manufacturing tolerances	8.02mdeg
Motor Gearbox	Backlash	No data

**Table 4:** This table highlights the individual pointing errors of each axis as well as the overall pointing error. Both the RMSE ($\epsilon_{RMSE}$) and the maximum pointing error ($\epsilon_{max}$) are shown.

	Pan Axis	Tilt Axis	Total
$\epsilon_{RMSE}$	2.801 mdeg	3.671 mdeg	6.179 mdeg
$\epsilon_{max}$	9.355 mdeg	18.278 mdeg	18.854 mdeg

**Table 5:** Project cost analysis including purchased components and materials. As manufacturing took place in-house, this is not listed here. Some components were already available in the workshop and their original price is not known; the respective costs were estimated.

Component	Amount	Price	Total
Dynamixel xl430-w250-t	2	$48.90	$97.80
Motor controller (estimated)	1	$10.00	$10.00
SKF W617/5 Bearing	8	$5.39	$43.08
SKF WBB1-8705 Bearing	4	$5.39	$21.54
igus JSM-1618-10 linear guide	1	$3.91	$3.91
igus LSM-1012-20 linear guide	4	$4.31	$17.23
igus sliding axis 16 mm	1	$43.08	$43.08
igus sliding axis 10 mm	4	$32.31	$129.24
Ball screws (estimated)	2	$200.00	$400.00
Aluminium frame and fastenerys			$179.68
3D print PETG filament			$10.77
Aluminium 6061 blocks			$53.85
Total			$1010.18

## Data Availability

The data presented in this study are openly available and can be found here: https://projects.asl.ethz.ch/datasets/doku.php?id=high_precision_gimbal (accessed on 31 December 2021).

## Abbreviations

The following abbreviations are used in this manuscript:

IMU	Inertial Measurement Unit
DoF	Degrees of Freedom
FoV	Field of View
RMSE	Root Mean Square Error
LiDAR	Light Detection and Ranging
MEMS	Micro-Electromechanical Systems
ETCS	European Train Control System
RoI	Region of Interest
LROD	Long-Range Obstacle Detection
CAD	Computer-Aided Design
CNC	Computer Numerical Control
USB	Universal Serial Bus
SDK	Software Development Kit
DC	Direct Current
ROS	Robot Operating System
PID	Proportional–Integral–Derivative
FEM	Finite Element Method
RGB	Red, Green and Blue

## References

[1] Stanley P. (2011). ETCS for Engineers.

[2] Barney D., Haley D., Nikandros G. (2001). Calculating Train Braking Distance. Conferences in Research and Practice in Information Technology Series.

[3] Ristić-Durrant D., Franke M., Michels K. (2021). A Review of Vision-Based On-Board Obstacle Detection and Distance Estimation in Railways. Sensors.

[4] Fel L., Zinner C., Kadiofsky T., Pointner W., Weichselbaum J., Reisner C. (2018). ODAS—An Anti-Collision Assistance System for Light Rail Vehicles and Further Development. Proceedings of the 7th Transport Research Arena TRA.

[5] Poulton C.V., Byrd M.J., Russo P., Timurdogan E., Khandaker M., Vermeulen D., Watts M.R. (2019). Long-Range LiDAR and Free-Space Data Communication With High-Performance Optical Phased Arrays. IEEE J. Sel. Top. Quantum Electron..

[6] Kroemer Elbert K.E., Kroemer H.B., Kroemer Hoffman A.D. (2018). Size and Mobility of the Human Body. Ergonomics.

[7] He D., Zou Z., Chen Y., Liu B., Yao X., Shan S. (2021). Obstacle Detection of Rail Transit Based on Deep Learning. Measurement.

[8] Kapoor R., Goel R., Sharma A. (2020). Deep Learning Based Object and Railway Track Recognition Using Train Mounted Thermal Imaging System. J. Comput. Theor. Nanosci..

[9] Mukojima H., Deguchi D., Kawanishi Y., Ide I., Murase H., Ukai M., Nagamine N., Nakasone R. (2016). Moving Camera Background-Subtraction for Obstacle Detection on Railway Tracks. Proceedings of the 2016 IEEE International Conference on Image Processing (ICIP).

[10] Uribe J.A., Fonseca L., Vargas J.F. (2012). Video Based System for Railroad Collision Warning. Proceedings of the 2012 IEEE International Carnahan Conference on Security Technology (ICCST).

[11] Nassu B.T., Ukai M. (2010). Automatic Recognition of Railway Signs Using SIFT Features. Proceedings of the 2010 IEEE Intelligent Vehicles Symposium.

[12] Manikandan R., Balasubramanian M., Palanivel S. (2017). Vision based obstacle detection on railway track. Int. J. Pure Appl. Math..

[13] Wang Z., Wu X., Yu G., Li M. (2018). Efficient Rail Area Detection Using Convolutional Neural Network. IEEE Access.

[14] Nakasone R., Nagamine N., Ukai M., Mukojima H., Deguchi D., Murase H. (2017). Frontal Obstacle Detection Using Background Subtraction and Frame Registration. Q. Rep. RTRI.

[15] Saika S., Takahashi S., Takeuchi M., Katto J. (2016). Accuracy Improvement in Human Detection Using HOG Features on Train-Mounted Camera. Proceedings of the 2016 IEEE 5th Global Conference on Consumer Electronics.

[16] Ye T., Wang B., Song P., Li J. (2018). Automatic Railway Traffic Object Detection System Using Feature Fusion Refine Neural Network under Shunting Mode. Sensors.

[17] Fonseca Rodriguez L.A., Uribe J.A., Vargas Bonilla J.F. (2012). Obstacle Detection over Rails Using Hough Transform. Proceedings of the 2012 XVII Symposium of Image, Signal Processing, and Artificial Vision (STSIVA).

[18] Yu M., Yang P., Wei S. (2018). Railway Obstacle Detection Algorithm Using Neural Network. AIP Conf. Proc..

[19] Ye T., Zhang X., Zhang Y., Liu J. (2021). Railway Traffic Object Detection Using Differential Feature Fusion Convolution Neural Network. IEEE Trans. Intell. Transp. Syst..

[20] Xu Y., Gao C., Yuan L., Tang S., Wei G. (2019). Real-Time Obstacle Detection Over Rails Using Deep Convolutional Neural Network. Proceedings of the 2019 IEEE Intelligent Transportation Systems Conference (ITSC).

[21] Li J., Zhou F., Ye T. (2018). Real-World Railway Traffic Detection Based on Faster Better Network. IEEE Access.

[22] RailVision.IO (2021). Technical Report, RailVision.IO. https://railvision.io.

[23] RailwayPro (2020). Obstacle Detection Systems for SBB Cargo Shunting Locomotives. Article, RailwayPro..

[24] Ristić-Durrant D., Haseeb M.A., Franke M., Banić M., Simonović M., Stamenković D., Bernardi S., Vittorini V., Flammini F., Nardone R., Marrone S., Adler R., Schneider D., Schleiß P., Nostro N., Løvenstein Olsen R. (2020). Artificial Intelligence for Obstacle Detection in Railways: Project SMART and Beyond. Dependable Computing—EDCC 2020 Workshops.

[25] Kudinov I.A., Kholopov I.S. (2020). Perspective-2-Point Solution in the Problem of Indirectly Measuring the Distance to a Wagon. Proceedings of the 2020 9th Mediterranean Conference on Embedded Computing (MECO).

[26] Fioretti F., Ruffaldi E., Avizzano C.A. (2018). A Single Camera Inspection System to Detect and Localize Obstacles on Railways Based on Manifold Kalman Filtering. Proceedings of the 2018 IEEE 23rd International Conference on Emerging Technologies and Factory Automation (ETFA).

[27] Weichselbaum J., Zinner C., Gebauer O., Pree W. (2013). Accurate 3D-Vision-Based Obstacle Detection for an Autonomous Train. Comput. Ind..

[28] Zhou X., Guo B., Wei W., Zhuang S., Chu J., Pan J.W. (2018). Railway Clearance Intrusion Detection Method with Binocular Stereo Vision. Young Scientists Forum 2017.

[29] Chernov A., Butakova M., Guda A., Shevchuk P., Bernardi S., Vittorini V., Flammini F., Nardone R., Marrone S., Adler R., Schneider D., Schleiß P., Nostro N., Løvenstein Olsen R. (2020). Development of Intelligent Obstacle Detection System on Railway Tracks for Yard Locomotives Using CNN. Dependable Computing—EDCC 2020 Workshops.

[30] Ukai M. (2006). A New System for Detecting Obstacles in Front of a Train. Railw. Technol. Avalance.

[31] Ukai M., Nassu B.T., Nagamine N., Watanabe M., Inaba T. Obstacle Detection on Railway Track by Fusing Radar and Image Sensor. Proceedings of the 9th World Congress on Railway Research (WCRR).

[32] Nassu B.T., Ukai M. (2012). A Vision-Based Approach for Rail Extraction and Its Application in a Camera Pan–Tilt Control System. IEEE Trans. Intell. Transp. Syst..

[33] Ruder M., Mohler N., Ahmed F. (2003). An Obstacle Detection System for Automated Trains. Proceedings of the IEEE IV2003 Intelligent Vehicles Symposium.

[34] Pavlović M., Ćirić I., Nikolić V., Simonović M., Stevanović J. Thermal image processing for autonomous train operation obstacle detection system. Proceedings of the XXVII MNTK “ADP-2018”.

[35] Pavlovic M., Ciric I., Ristic-Durrant D., Nikolic V., Simonovic M., Ciric M., Banic M. (2018). Advanced Thermal Camera Based System for Object Detection on Rail Tracks. Therm. Sci..

[36] Haseeb M.A., Ristić-Durrant D., Gräser A. Long-Range Obstacle Detection from a Monocular Camera. Proceedings of the ACM Computer Science in Cars Symposium (CSCS).

[37] Ristić-Durrant D., Haseeb M.A., Emami D., Gräser A. (2018). Multimodal Sensor Fusion for Reliable Detection of Obstacles on Railway Tracks. J. Mechatron. Autom. Identif. Technol..

[38] Ristić-Durrant D., Haseeb M.A., Banić M., Stamenković D., Simonović M., Nikolić D. (2021). SMART On-Board Multi-Sensor Obstacle Detection System for Improvement of Rail Transport Safety. Proc. Inst. Mech. Eng. Part F J. Rail Rapid Transit.

[39] Ristić-Durrant D., Haseeb M.A., Banić M., Stamenković D., Simonović M., Miltenović A., Nikolić V., Nikolić D. Obstacle detection for railways: Lessons learned from project smart. Proceedings of the XIX International Scientific-Expert Conference on Railways—RAILCON 2020.

[40] Ristić-Durrant D., Ćirić I., Simonović M., Nikolić V., Leu A., Brindić B. Towards autonomous obstacle detection in freight railway. Proceedings of the XVII International Scientific-Expert Conference on Railways.

[41] Zhangyu W., Guizhen Y., Xinkai W., Haoran L., Da L. (2021). A Camera and LiDAR Data Fusion Method for Railway Object Detection. IEEE Sens. J..

[42] Mockel S., Scherer F., Schuster P. (2003). Multi-Sensor Obstacle Detection on Railway Tracks. Proceedings of the IEEE IV2003 Intelligent Vehicles Symposium.

[43] Yokoyama H., Date H., Kanai S., Takeda H. (2013). Detection and Classification of Pole-like Objects from Mobile Laser Scanning Data of Urban Environments. Int. J. CAD/CAM.

[44] Karaduman M. Image Processing Based Obstacle Detection with Laser Measurement in Railways. Proceedings of the 2017 10th International Conference on Electrical and Electronics Engineering (ELECO).

[45] Yamashita H., Iida Y., Nakamoto J., Koyama Y., Sato M. (1996). Development of Railway Obstacle Detection System.

[46] Tan W., Yan B., Bare B. Feature Super-Resolution: Make Machine See More Clearly. Proceedings of the IEEE Conference on Computer Vision and Pattern Recognition (CVPR).

[47] Ren Y., Zhu C., Xiao S. (2018). Small Object Detection in Optical Remote Sensing Images via Modified Faster R-CNN. Appl. Sci..

[48] Li Y., Shi T., Zhang Y., Chen W., Wang Z., Li H. (2021). Learning Deep Semantic Segmentation Network under Multiple Weakly-Supervised Constraints for Cross-Domain Remote Sensing Image Semantic Segmentation. ISPRS J. Photogramm. Remote Sens..

[49] Yang X., Wu W., Liu K., Kim P.W., Sangaiah A.K., Jeon G. (2018). Long-Distance Object Recognition With Image Super Resolution: A Comparative Study. IEEE Access.

[50] Li K., Wan G., Cheng G., Meng L., Han J. (2020). Object Detection in Optical Remote Sensing Images: A Survey and a New Benchmark. ISPRS J. Photogramm. Remote Sens..

[51] James J., Ford J.J., Molloy T.L. (2018). Learning to Detect Aircraft for Long-Range Vision-Based Sense-and-Avoid Systems. IEEE Robot. Autom. Lett..

[52] van den Hoogen B., Uijens W., den Hollander R.J.M., Huizinga W., Dijk J., Schutte K., Dijk J. (2020). Long-Range Person and Vehicle Detection. Artificial Intelligence and Machine Learning in Defense Applications II.

[53] Williamson T., Thorpe C. (1999). A Trinocular Stereo System for Highway Obstacle Detection. Proceedings of the 1999 IEEE International Conference on Robotics and Automation (Cat. No. 99CH36288C).

[54] Pinggera P., Franke U., Mester R. (2015). High-Performance Long Range Obstacle Detection Using Stereo Vision. Proceedings of the 2015 IEEE/RSJ International Conference on Intelligent Robots and Systems (IROS).

[55] Wang H., Mou X., Mou W., Yuan S., Ulun S., Yang S., Shin B.S. (2015). Vision Based Long Range Object Detection and Tracking for Unmanned Surface Vehicle. Proceedings of the 2015 IEEE 7th International Conference on Cybernetics and Intelligent Systems (CIS) and IEEE Conference on Robotics, Automation and Mechatronics (RAM).

[56] Zhang K., Xie J., Snavely N., Chen Q. (2020). Depth Sensing Beyond LiDAR Range. Proceedings of the 2020 IEEE/CVF Conference on Computer Vision and Pattern Recognition (CVPR).

[57] Dickmanns E.D. An Advanced Vision System For Ground Vehicles. Proceedings of the 1st International Workshop on In-Vehicle Cognitive Computer Vision Systems (IVCCVS).

[58] Kendall A., Martirosyan H., Dasgupta S., Henry P., Kennedy R., Bachrach A., Bry A. (2017). End-to-End Learning of Geometry and Context for Deep Stereo Regression. arXiv.

[59] Tonioni A., Tosi F., Poggi M., Mattoccia S., Di Stefano L. (2019). Real-Time Self-Adaptive Deep Stereo. arXiv.

[60] Yang G., Zhao H., Shi J., Deng Z., Jia J. (2018). SegStereo: Exploiting Semantic Information for Disparity Estimation. arXiv.

[61] Perrollaz M., Labayrade R., Royere C., Hautiere N., Aubert D. (2006). Long Range Obstacle Detection Using Laser Scanner and Stereovision. Proceedings of the 2006 IEEE Intelligent Vehicles Symposium.

[62] Labayrade R., Gruyer D., Royere C., Perrollaz M., Aubert D., Kolski S. (2007). Obstacle Detection Based on Fusion Between Stereovision and 2D Laser Scanner. Mobile Robots: Perception &amp Navigation.

[63] Alessandretti G., Broggi A., Cerri P. (2007). Vehicle and Guard Rail Detection Using Radar and Vision Data Fusion. IEEE Trans. Intell. Transp. Syst..

[64] Candan C., Tiken M., Berberoglu H., Orhan E., Yeniay A. (2021). Experimental Study on Km-Range Long-Distance Measurement Using Silicon Photomultiplier Sensor with Low Peak Power Laser Pulse. Appl. Sci..

[65] Villgrattner T., Ulbrich H. (2011). Design and Control of a Compact High-Dynamic Camera-Orientation System. IEEE/ASME Trans. Mechatron..

[66] Hudgens J.M., Cawthon G.M. Extreme accuracy tracking gimbal for radome measurements. Proceedings of the Antenna Measurement Techniques Association 25th Annual Meeting & Symposium (AMTA-03).

[67] Li H., Han K., Wang X., He S., Wu Q., Xu Z. (2019). A Compact and Lightweight Two-Dimensional Gimbal for Inter-Satellite Laser Communication Applications. Opt. Express.

[68] Talmor A.G., Harding H., Chen C.C., Hemmati H., Boroson D.M. (2016). Two-Axis Gimbal for Air-to-Air and Air-to-Ground Laser Communications. Free-Space Laser Communication and Atmospheric Propagation XXVIII.

[69] Bajaj N.M., Spiers A.J., Dollar A.M. (2015). State of the Art in Prosthetic Wrists: Commercial and Research Devices. Proceedings of the 2015 IEEE International Conference on Rehabilitation Robotics (ICORR).

[70] Ferris M., Phillips N. The use and advancement of an affordable, adaptable antenna pointing mechanism. Proceedings of the 14th European Space Mechanisms & Tribology Symposium.

[71] Jiang X., Fan D., Fan S., Xie X., Chen N. (2021). High-Precision Gyro-Stabilized Control of a Gear-Driven Platform with a Floating Gear Tension Device. Front. Mech. Eng..

[72] Shaffer R., Karpenko M., Gong Q. (2018). Robust Control of a Flexible Double Gimbal Mechanism. Proceedings of the 2018 Annual American Control Conference (ACC).

[73] Bai Z.F., Zhao J.J., Chen J., Zhao Y. (2018). Design Optimization of Dual-Axis Driving Mechanism for Satellite Antenna with Two Planar Revolute Clearance Joints. Acta Astronaut..

[74] Kesner J.E., Hinrichs K.M., Narkewich L.E., Stephens T., Hemmati H., Boroson D.M. (2015). Compact Optical Gimbal as a Conformal Beam Director for Large Field-of-Regard Lasercom Applications. Free-Space Laser Communication and Atmospheric Propagation XXVII.

[75] Kaymak Y., Rojas-Cessa R., Feng J., Ansari N., Zhou M., Zhang T. (2018). A Survey on Acquisition, Tracking, and Pointing Mechanisms for Mobile Free-Space Optical Communications. IEEE Commun. Surv. Tutor..

[76] Shi Z., Song H., Chen H., Sun Y. (2018). Research on Measurement Accuracy of Laser Tracking System Based on Spherical Mirror with Rotation Errors of Gimbal Mount Axes. Meas. Sci. Rev..

[77] Truong S.N., Kieffer J., Zelinsky A. A Cable-Driven Pan-Tilt Mechanism’ for Active Vision. Proceedings of the Australian Conference on Robotics and Automation.

[78] Qiu C., Wu Z., Kong S., Yu J. (2021). An Underwater Micro Cable-Driven Pan-Tilt Binocular Vision System With Spherical Refraction Calibration. IEEE Trans. Instrum. Meas..

[79] Kim M.D., Ueda J. (2015). Dynamics-Based Motion de-Blurring for a PZT-Driven, Compliant Camera Orientation Mechanism. Int. J. Robot. Res..

[80] Osborne J., Hicks G., Fuentes R. (2008). Global Analysis of the Double-Gimbal Mechanism. IEEE Control Syst..

[81] Pertile M., Debei S., Zaccariotto M. (2009). Accuracy Analysis of a Pointing Mechanism for Communication Applications. IEEE Trans. Instrum. Meas..

[82] Rosheim M.E., Sauter G.F., Ricklin J.C., Voelz D.G. (2002). New High-Angulation Omni-Directional Sensor Mount. Free-Space Laser Communication and Laser Imaging II.

[83] Nikulin V.V. (2005). Advanced Lyapunov Control of a Novel Laser Beam Tracking System. Opt. Eng..

[84] Sofka J., Skormin V.A., Nikulin V.V., Nicholson D.J., Rosheim M., Voelz D.G., Ricklin J.C. (2004). New Generation of Gimbals Systems for Laser Positioning Applications. Free-Space Laser Communication and Active Laser Illumination III.

[85] Sofka J., Skormin V., Nikulin V., Nicholson D. (2006). Omni-Wrist III—A New Generation of Pointing Devices. I. Laser Beam Steering Devices—Mathematical Modeling. IEEE Trans. Aerosp. Electron. Syst..

[86] Sofka J., Skormin V., Nikulin V., Nicholson D. (2006). Omni-Wrist III–A New Generation of Pointing Devices. II. Gimbals Systems-Control. IEEE Trans. Aerosp. Electron. Syst..

[87] Sofka J., Skormin V. (2006). Integrated Approach to Electromechanical Design of a Digitally Controlled High Precision Actuator for Aerospace Applications. Proceedings of the 2006 IEEE Conference on Computer Aided Control System Design, 2006 IEEE International Conference on Control Applications, 2006 IEEE International Symposium on Intelligent Control.

[88] Sofka J., Nikulin V., Skormin V., Hughes D., Legare D. (2009). Laser Communication Between Mobile Platforms. IEEE Trans. Aerosp. Electron. Syst..

[89] Nikulin V.V. (2008). Agile Acousto-Optic Tracking System for Free-Space Optical Communications. Opt. Eng..

[90] Oleski P.J., Dorrian K.W., Busch T.E., Mecherle G.S. (1994). Nonmechanical Laser Beam Steering/Beam Spoiling Methods for Intersatellite Cross Links. SPIE 2123, Free-Space Laser Communication Technologies VI.

[91] Nikulin V.V. (2001). Modeling of an Acousto-Optic Laser Beam Steering System Intended for Satellite Communication. Opt. Eng..

[92] Sofka J., Nikulin V.V., Skormin V.A., Nicholson D.J., Voelz D.G., Ricklin J.C. (2004). Hybrid Laser Beam Steerer for Laser Communications Applications. Free-Space Laser Communication and Active Laser Illumination III.

[93] Villgrattner T., Schneider E., Andersch P., Ulbrich H. (2011). Compact High Dynamic 3 DoF Camera Orientation System: Development and Control. J. Syst. Des. Dyn..

[94] Antonello R., Branz F., Sansone F., Cenedese A., Francesconi A. (2021). High-Precision Dual-Stage Pointing Mechanism for Miniature Satellite Laser Communication Terminals. IEEE Trans. Ind. Electron..

[95] Carricato M., Parenti-Castelli V. (2004). A Novel Fully Decoupled Two-Degrees-of-Freedom Parallel Wrist. Int. J. Robot. Res..

[96] Villgrattner T., Ulbrich H. (2010). Optimization and Dynamic Simulation of a Parallel Three Degree-of-Freedom Camera Orientation System. Proceedings of the 2010 IEEE/RSJ International Conference on Intelligent Robots and Systems.

[97] Villgrattner T., Ulbrich H. (2008). Piezo-Driven Two-Degree-of-Freedom Camera Orientation System. Proceedings of the 2008 IEEE International Conference on Industrial Technology.

[98] Cannata G., Maggiali M., de Pina Filho A.C. (2007). Design of a Humanoid Robot Eye. Humanoid Robots: New Developments.

[99] Cannata G., Maggiali M. (2008). Models for the Design of Bioinspired Robot Eyes. IEEE Trans. Robot..

[100] Wang X.Y., Zhang Y., Fu X.J., Xiang G.S. (2008). Design and Kinematic Analysis of a Novel Humanoid Robot Eye Using Pneumatic Artificial Muscles. J. Bionic Eng..

[101] Lee Y.C., Lan C.C., Chu C.Y., Lai C.M., Chen Y.J. (2013). A Pan–Tilt Orienting Mechanism With Parallel Axes of Flexural Actuation. IEEE/ASME Trans. Mechatron..

[102] Zelenika S., Rohrer M., Rossetti D. Ultra-High Precision Gimbal-Mount for Optical Elements. Proceedings of the 5th EUSPEN International Conference.

[103] Huajun H., Bo P., Haowei W., Haiyuan W., Fan Z., Jing S. (2020). Design and Analysis of A 2-DOF Parallel Mechanism for Space Large Deployable Antenna. Proceedings of the 2020 4th International Conference on Vision, Image and Signal Processing.

[104] Tsujita T., Konno A., Uchiyama M. (2005). Design and Development of a High Speed Binocular Camera Head. Proceedings of the 2005 IEEE International Conference on Robotics and Automation.

[105] Liu W.W., Yang J.F., Wang Y.H., Cai M.X., Hu Y., Dong C.J. (2020). Kinematic Calibration of a Gough-Stewart Based High-Precision Pointing Mechanism. Proceedings of the 2020 International Symposium on Autonomous Systems (ISAS).

[106] Wang Y.H., Yang J.F., Cai M.X., Huang Q., Mo W.A., Wang W.H. (2020). Kinematic Modeling and Simulation of a Gough-Stewart Based High-Precision Pointing Mechanism. Proceedings of the 2020 International Symposium on Autonomous Systems (ISAS).

[107] Chen H.J., Hospodar E., Agrawal B. (2004). Development of a Hexapod Laser-Based Metrology System for Finer Optical Beam Pointing Control. Proceedings of the 22nd AIAA International Communications Satellite Systems Conference & Exhibit 2004 (ICSSC).

[108] McInroy J., O’Brien J., Neat G. (1999). Precise, Fault-Tolerant Pointing Using a Stewart Platform. IEEE/ASME Trans. Mechatron..

[109] Chen H.J., Bishop R., Agrawal B. (2003). Payload Pointing and Active Vibration Isolation Using Hexapod Platforms. Proceedings of the 44th AIAA/ASME/ASCE/AHS/ASC Structures, Structural Dynamics, and Materials Conference.

[110] Hospodar E.J. (2003). A Laser Metrology System for Precision Pointing. Ph.D. Thesis.

[111] Geijo E.M., Casalta J.M., Canchado M., San Andrés M., Brú R., García H., Tomàs A., Zago L., Jeffers P., Atad-Ettedgui E., Antebi J., Lemke D. (2006). VISTA Secondary Mirror Drive Performance and Test Results. Proceedings of the SPIE—The International Society for Optical Engineering.

[112] Dong W., Sun L., Du Z. (2007). Design of a Precision Compliant Parallel Positioner Driven by Dual Piezoelectric Actuators. Sens. Actuators A Phys..

[113] Gosselin C.M., Cloutier C., Rancourt D. Kinematic Analysis of Spherical Two-Degree-of-Freedom Parallel Manipulators. In Proceeding of the International Design Engineering Technical Conferences and Computers and Information in Engineering Conference.

[114] Majid M.Z.A., Huang Z., Yao Y.L. (2000). Workspace Analysis of a Six-Degrees of Freedom, Three-Prismatic- Prismatic-Spheric-Revolute Parallel Manipulator. Int. J. Adv. Manuf. Technol..

[115] Koseki Y., Arai T., Sugimoto K., Takatuji T., Goto M. (1998). Design and Accuracy Evaluation of High-Speed and High Precision Parallel Mechanism. Proceedings of the 1998 IEEE International Conference on Robotics and Automation (Cat. No. 98CH36146).

[116] Li G., Huang H., Li B. (2021). Design and Optimization of a 6-DOF Singularity-Free Parallel Manipulator. Int. J. Robot. Autom. Technol..

[117] Di Gregorio R. (2001). A New Parallel Wrist Using Only Revolute Pairs: The 3-RUU Wrist. Robotica.

[118] Stigger T., Siegele J., Scharler D.F., Pfurner M., Husty M.L. (2019). Analysis of a 3-RUU Parallel Manipulator. Mech. Mach. Theory.

[119] Zierer J.J., Beno J.H., Weeks D.A., Soukup I.M., Good J.M., Booth J.A., Hill G.J., Rafal M.D., Stepp L.M., Gilmozzi R., Hall H.J. (2012). Design, Testing, and Installation of a High-Precision Hexapod for the Hobby-Eberly Telescope Dark Energy Experiment (HETDEX). Ground-Based and Airborne Telescopes IV.

[120] Di Gregorio R. (2001). Kinematics of a New Spherical Parallel Manipulator with Three Equal Legs: The 3-URC Wrist. J. Robot. Syst..

[121] Hu Y., Wan Z., Yao J., Zhang J. (2009). Singularity and Kinematics Analysis for a Class of PPUU Mobile Parallel Robots. Proceedings of the 2009 IEEE International Conference on Robotics and Biomimetics (ROBIO).

[122] Huynh P., Herve J.M. (2005). Equivalent Kinematic Chains of Three Degree-of-Freedom Tripod Mechanisms With Planar-Spherical Bonds. J. Mech. Des..

[123] McInroy J. (2002). Modeling and Design of Flexure Jointed Stewart Platforms for Control Purposes. IEEE/ASME Trans. Mechatron..

[124] Zhang G., Guo J., Hou Y., Zeng D. (2021). Analysis of the PU-2UPS Antenna Parallel Mechanism. J. Mech. Sci. Technol..

[125] Du Z., Shi R., Dong W. (2014). A Piezo-Actuated High-Precision Flexible Parallel Pointing Mechanism: Conceptual Design, Development, and Experiments. IEEE Trans. Robot..

[126] Burger F., Eder J. High-Precision Pointing Device for the LASCO Instrument on SOHO. Proceedings of the Sixth European Space Mechanisms and Tribology Symposium.

[127] Henein S., Schwab P., Kjelberg I., Giriens L., Sa C., Greger R., Langer U., Walz S. Design and Development of the Point-Ahead Angle Mechanism for the Lase Interferometer Space Antenna (LISA). Proceedings of the 13th European Space Mechanisms & Tribology Symposium.

[128] Bandera P. A Fine Pointing Mechanism for Intersatellite Laser Communication. European Space Agency-Publications-ESA SP.. https://www.esmats.eu/esmatspapers/pastpapers/pdfs/1999/bandera.pdf.

[129] Hafez M., Sidler T., Salathé R., Jansen G., Compter J. (2000). Design, Simulations and Experimental Investigations of a Compact Single Mirror Tip/Tilt Laser Scanner. Mechatronics.

[130] Aoki K., Yanagita Y., Kuroda H., Shiratama K., Voelz D.G., Ricklin J.C. (2004). Wide-Range Fine Pointing Mechanism for Free-Space Laser Communications. Free-Space Laser Communication and Active Laser Illumination III.

[131] Harvison D.A., Hardy B., Bely P.Y., Breckinridge J.B. (1996). Precision Pointing Mechanism for Laser Communication Mission. Space Telescopes and Instruments IV.

[132] Chakraborty P., Behdinan K., Tabarrok B. (1999). A tip-tilt adaptive optics system for amateur astronomers and optimum placement of actuators. J. Sound Vib..

[133] Shao B., Chen L., Rong W., Ru C., Xu M. (2009). Modeling and Design of a Novel Precision Tilt Positioning Mechanism for Inter-Satellite Optical Communication. Smart Mater. Struct..

[134] Gosselin C., Hamel J.F. (1994). The Agile Eye: A High-Performance Three-Degree-of-Freedom Camera-Orienting Device. Proceedings of the 1994 IEEE International Conference on Robotics and Automation.

[135] Merriam E.G., Jones J.E., Magleby S.P., Howell L.L. (2013). Monolithic 2 DOF Fully Compliant Space Pointing Mechanism. Mech. Sci..

[136] Zoppi M., Molfino R. (2007). ArmillEye: Flexible Platform for Underwater Stereo Vision. J. Mech. Des..

[137] Gosselin C., Caron F. (1999). Two Degree-of-Freedom Spherical Orienting Device. U.S. Patent.

[138] Gosselin C.M., Lavoie E. (1993). On the Kinematic Design of Spherical Three-Degree-of- Freedom Parallel Manipulators. Int. J. Robot. Res..

[139] Gogu G. (2005). Fully-Isotropic Over-Constrained Parallel Wrists with Two Degrees of Freedom. Proceedings of the 2005 IEEE International Conference on Robotics and Automation.

[140] Li W., He K., Qu Y., Zhang J., Du R. Hemisphere, a fully decoupled parallel 2-DOF spherical mechanism. Proceedings of the 7th WSEAS International Conference on Robotics, Control & Manufacturing Technology.

[141] Leguay-Durand S., Reboulet C. (1997). Optimal Design of Aredundant Spherical Parallel Manipulator. Robotica.

[142] Herve J. (2006). Uncoupled Actuation of Pan-Tilt Wrists. IEEE Trans. Robot..

[143] Ma Y., Islam S., Pan Y.J. (2011). Electrostatic Torsional Micromirror With Enhanced Tilting Angle Using Active Control Methods. IEEE/ASME Trans. Mechatron..

[144] Bassett K., Hammond M., Smoot L. (2009). A Fluid-Suspension, Electromagnetically Driven Eye with Video Capability for Animatronic Applications. Proceedings of the 2009 9th IEEE-RAS International Conference on Humanoid Robots.

[145] Bederson B., Wallace R., Schwartz E. (1993). Control and Design of the Spherical Pointing Motor. Proceedings of the IEEE International Conference on Robotics and Automation.

[146] Bederson B., Wallace R., Schwartz E. (1994). A Miniature Pan-Tilt Actuator: The Spherical Pointing Motor. IEEE Trans. Robot. Autom..

[147] Bederson B.B., Wallace R.S., Schwartz E. (1995). A Miniaturized Space-Variant Active Vision System: Cortex-I. Mach. Vis. Appl..

[148] Lee J.-S., Kim D.-K., Baek S.-W., Rhyu S., Kwon B. (2008). Newly Structured Double Excited Two-Degree-of-Freedom Motor for Security Camera. IEEE Trans. Magn..

[149] Hoshina M., Mashimo T., Toyama S. (2009). Development of Spherical Ultrasonic Motor as a Camera Actuator for Pipe Inspection Robot. Proceedings of the 2009 IEEE/RSJ International Conference on Intelligent Robots and Systems.

[150] Fisk J.W., Rue A.K. (1966). Confidence Limits for the Pointing Error of Gimbaled Sensors. IEEE Trans. Aerosp. Electron. Syst..

[151] Sweeney M.N., Erdelyi E., Ketabchi M., Kent B., Hatheway A.E. (2003). Design Considerations for Optical Pointing and Scanning Mechanisms. Optomechanics 2003.

[152] DJI RS 2—Specifications—DJI. https://www.dji.com/rs-2/specs.

[153] DJI Ronin 2—Specifications, FAQs, Videos, Tutorials, Manuals, DJI GO—DJI. https://www.dji.com/ronin-2/info.

[154] GREMSY T3V3—Gremsy. https://gremsy.com/gremsy-t3v3-store#.

[155] Motorized Goniometers (Gimbal Mounts) for Large Square Optics—Custom Engineering—Catalog—Opto-Mechanical Products—Standa. https://www.standa.lt/products/catalog/custom_engineering?item=570.

[156] Mōvi Pro. https://freeflysystems.com/movi-pro.

[157] AMG Direct-Drive Gimbals. https://www.aerotech.com/product/gimbals-optical-mounts/amg-direct-drive-gimbals/.

[158] FG-URS Motorized Full Gimbal System. https://www.newport.com/p/FG-URS.

[159] Motorized Two-Axis Pan-Tilt Stage, 60 Mm Diameter). http://oesincorp.com/motorized-multi-axis-stages/motorized-two-axis-pan-tilt-stage-60mm-diameter.htm.

[160] FLIR PTU-5 Pan/Tilt for Top-Mounted Payloads up to 5 lbs|Teledyne FLIR. https://www.flir.eu/products/ptu-5/.

[161] (2021). Robotis. DYNAMIXEL XL430-W250-T. Datasheet, Robotis. https://www.robotis.us/dynamixel-xl430-w250-t/.

[162] Son W., Dynamixel SDK (2021). Robotis. https://github.com/ROBOTIS-GIT/DynamixelSDK.

[163] Hale L. (1999). Principles and Techniques for Designing Precision Machines. Ph.D. Thesis.

[164] Baumer (2021). Encoders without Bearings—Absolute/HDmag Flex MQR 3000F. Datasheet MQR 3000F, Baumer..

[165] ANYbotics (2016). ANYdrive—Modular Joint Units. Technical Report, ANYbotics..

[166] Hwang Y.K., Lee C.M. (2010). A Review on the Preload Technology of the Rolling Bearing for the Spindle of Machine Tools. Int. J. Precis. Eng. Manuf..

[167] Selzer D.R., Iglide® J/Iglidur® J Datasheet (2010). Datasheet F-2-0034-A, IGUS. https://www.igus.com/ContentData/Products/Downloads/iglide_J_product_data_sheet.pdf.

[168] Selzer D.R., Iglidur® W300 Datasheet (2013). Datasheet F-2-0034-A, Igus. https://www.igus.com/ContentData/Products/Downloads/iglidur_W300_product_data_sheet2013.pdf.

[169] Lin C.Y., Hung J.P., Lo T.L. (2010). Effect of Preload of Linear Guides on Dynamic Characteristics of a Vertical Column–Spindle System. Int. J. Mach. Tools Manuf..

[170] SKF (2021). W 617/5 Rillenkugellager. Datasheet W 617/5, SKF..

[171] SKF (2021). WBB1-8705 Rillenkugellager. Datasheet WBB1-8705, SKF..

[172] Smith S.T. (2003). Foundations of Ultra-Precision Mechanism Design.

[173] Baumer (2019). EAM580-B—Absolute Encoders. Technical Report, Baumer..

[174] AMS (2016). AS5601—12-Bit Programmable Contactless Encoder. Datasheet, AMS..

[175] Quigley M., Gerkey B., Conley K., Faust J., Foote T., Leibs J., Berger E., Wheeler R., Ng A. ROS: An Open-Source Robot Operating System. Proceedings of the ICRA Workshop on Open Source Software.

[176] Arduino S.r.l., Arduino ZERO (2021). Technical Report, Arduino S.r.l. https://www.arduino.cc/en/Main/ArduinoBoardZero&.

[177] Tschopp F., Riner M., Fehr M., Bernreiter L., Furrer F., Novkovic T., Pfrunder A., Cadena C., Siegwart R., Nieto J. (2020). VersaVIS: An Open Versatile Multi-Camera Visual-Inertial Sensor Suite. Sensors.

[178] Bouchier P., Purvis M., Ferguson M. Rosserial. https://github.com/ros-drivers/rosserial.

[179] VISHAY (2021). TCST2103—Transmissive Optical Sensor with Phototransistor Output. Datasheet 81147, VISHAY..

[180] Leica Leica_disto_d8_user_manual_767718_en.Pdf. https://shop.leica-geosystems.com/sites/default/files/2019-03/leica_disto_d8_user_manual_767718_en.pdf.

[181] Picotronic (2013). Dot Laser, Red, 650 Nm, 1 mW. Datasheet DA650-1-5(11x60), Picotronic..

[182] Wang S.M., Ehmann K. (1994). Compensation of Geometric and Quasi-Static Deformation Errors of a Multi-Axis Machine. Trans. NAMRI/SME.

